# Dual role of DR5 in death and survival signaling leads to TRAIL resistance in cancer cells

**DOI:** 10.1038/cddis.2017.423

**Published:** 2017-08-31

**Authors:** Yelyzaveta Shlyakhtina, Valeria Pavet, Hinrich Gronemeyer

**Affiliations:** 1 Institut de Génétique et de Biologie Moléculaire et Cellulaire (IGBMC), Equipe Labellisée Ligue Contre le Cancer, Centre National de la Recherche Scientifique UMR 7104, Institut National de la Santé et de la Recherche Médicale U964, University of Strasbourg, Illkirch, France

## Abstract

Besides its tumor-selective apoptotic activity, tumor necrosis factor-related apoptosis-inducing ligand (TRAIL) promotes pro-survival, proliferative or migratory signaling (NF-*κ*B, PI3K/Akt, MAPK and JNK; referred to as 'non-apoptotic' cascades). Indeed, apoptosis and non-apoptotic signaling can be activated in clonal populations of cancer cells in response to treatment and, as a result, only a part of the initial cellular population dies while a fraction survives and develops resistance to TRAIL-induced apoptosis (referred to as 'fractional survival'). Notably, the molecular characterization of the protein platforms streaming into tumoricidal *versus* tumor-promoting cascades that control fractional survival remained elusive. Here we demonstrate that, in the context of DR4–DR5–DcR2 hetero-oligomeric complexes, a single death receptor (DR5) suffices to assemble composite plasma membrane-proximal pro-apoptotic/pro-survival platforms that propagate TRAIL signaling to both death and survival pathways in clonal populations of cancer cells. Moreover, we show that while all members of TRAIL-induced complexes support survival, none of them acted exclusively pro-apoptotic. Indeed, key apoptotic proteins as FADD and procaspase-8 were also involved in transducing non-apoptotic signaling in response to this cytokine. Collectively, this study reveals the Janus faces of DR5, and the contributions of other death complex components in fractional survival that foster the generation of resistance. Our data highlight a new level of complexity in TRAIL signaling and point to an improved therapeutic rationale in view of hitherto disappointing results.

Tumor necrosis factor-related apoptosis-inducing ligand (TRAIL, TNFSF10, Apo2L), a member of the tumor necrosis factor family of cytokines, binds to the extracellular domains of four plasma membrane-bound receptors inducing their oligomerization. Two 'death receptors' (DR4/TRAILR1/TNFRSF10A and DR5/TRAILR2/TNFRSF10A)^1, 2, 3, 4^ possess a cytoplasmic 'death domain' that serves as a platform for recruitment of the adaptor protein FADD (Fas-associated protein with death domain) that in turn promotes binding of initiator procaspases (−8 and/or −10), thereby assembling a death inducing signaling complex (DISC) in response to TRAIL.^5, 6^ In contrast, two other receptors act as 'decoy receptors' as they bind TRAIL with a similar affinity but do not assemble a DISC (DcR1/TRAILR3/TNFRSF10C and DcR2/TRAILR4/TNFRSF10D).^4, 7, 8^ TRAIL-induced DISC assembly promotes the activation of initiator procaspases, thus triggering the death-executing cascade.^9, 10^ Notably, several factors regulate the final cell fate once TRAIL-induced signaling is triggered, including TRAIL binding to its decoy receptors that sequester the ligand from death receptors and/or hamper the assembly of an apoptosis-proficient complex by getting recruited to the DISC.^11, 12, 13, 14, 15, 16^ Furthermore, activation of initiator procaspases is also inhibited by recruitment of the non-functional procaspase homolog cellular Flice-like inhibitory protein (cFlip), leading to apoptosis suppression.^17, 18, 19, 20^ Finally, the levels of pro- and anti-apoptotic proteins that modulate the activity of executor caspases or mitochondrial activation also impact on the ultimate cell fate following TRAIL treatment.^21, 22^

Besides its apoptogenic action, TRAIL activates non-apoptotic cascades^23^ in non-tumorigenic scenarios, in populations of cancer cells fully resistant to TRAIL-induced cell death and in processes of fractional survival.^12, 24, 25, 26, 27, 28, 29, 30, 31^ In that regard, activation of TRAIL-induced non-apoptotic cascades has been ascribed to the formation of a signaling complex (referred to as 'secondary complex') composed of FADD, caspase-8, RIPK1 (receptor-interacting serine/threonine-protein kinase 1), TRAF2 (TNF receptor-associated factor 2) and NEMO/IKK (NF-*κ*-B essential modulator).^23^ Yet, it remains unclear whether this complex is indeed an entity entirely distinct from the DISC, as the subcellular compartment(s) where TRAIL-induced signaling complex(es) is/are formed and the role of individual TRAIL receptors in the assembly of such platform(s) are largely unknown. To address these issues, we have characterized here the complex(es) and dissected the role of individual TRAIL receptors and recruited proteins in apoptosis and non-apoptotic signaling in populations of cancer cells displaying fractional survival in response to TRAIL. We demonstrate that a single death receptor (DR5) and central DISC components (FADD and caspase-8) form the core of composite pro-apoptotic-pro-survival plasma membrane-proximal platform(s) that lead to apoptosis but concomitantly activate non-apoptotic cascades, thereby supporting establishment of reversible resistance in cancer cells. Our results are of major importance for the implementation of TRAIL-based cancer-selective therapies and provide a new rationale for the rather disapointing results obtained with TRAIL-therapeutics.^32, 33^

## Results

### TRAIL induces non-apoptotic pathways supporting fractional survival

**Figure 1:**
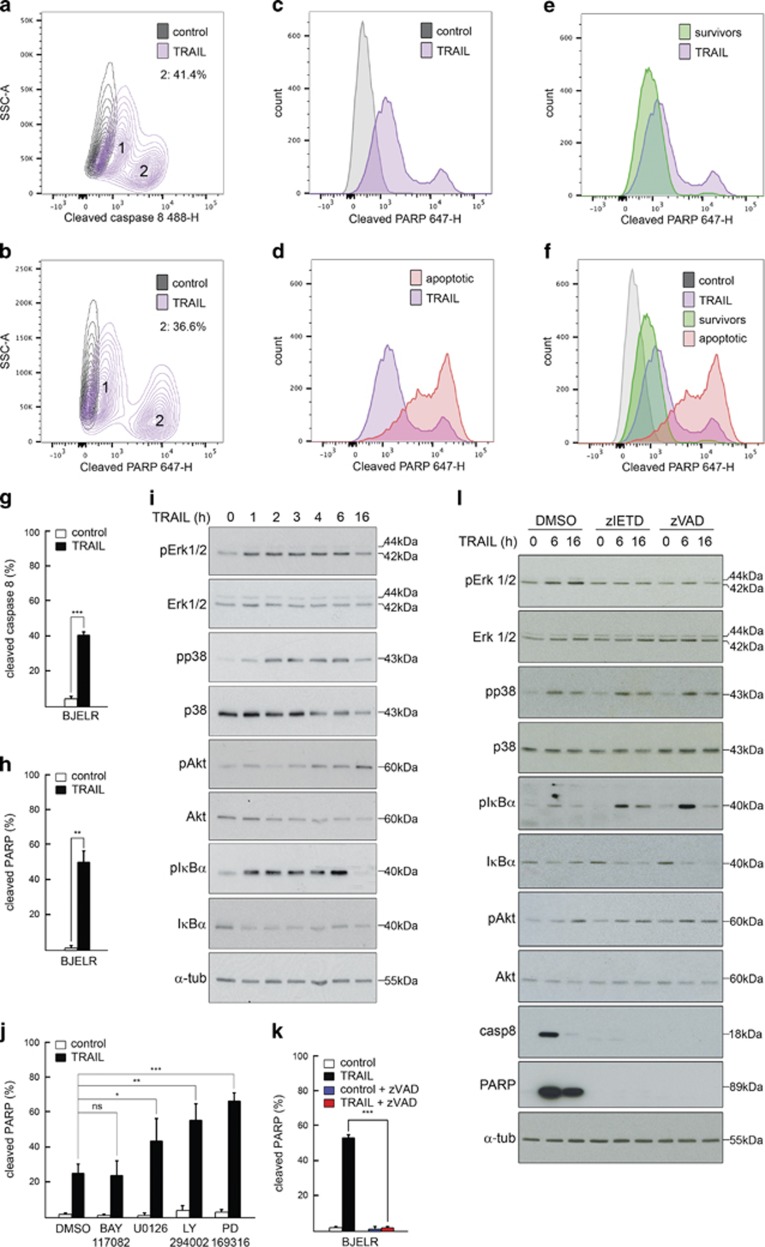
TRAIL induces the activation of apoptosis and non-apoptotic cascades leading to fractional survival. (**a**) Contour plot from flow cytometry assays assessing cleaved caspase-8 (p18) immunolabelling in populations of naïve (control) or TRAIL-treated cells (TRAIL, 3 h). Subpopulations displaying low (1) and high (2; percentage is depicted) levels of caspase-8 cleavage are indicated. (**b**) Experiment as in (**a**) but analyzing cleaved PARP immunolabelling (**c**–**f**) levels of cleaved PARP in clonal populations of naïve cells (control), the full population of cells treated 16 h with TRAIL (TRAIL) and subpopulations of apoptotic (apoptotic) or surviving (survivors) cells obtained after 16 h of treatment. (**a**–**f**) Images correspond to one representative experiment out of three independent biological replicates. (**g**–**h**) Percentage of cells with high levels of cleaved caspase-8 (**g**) or high levels of cleaved PARP (**h**) after TRAIL treatment (6 h, maximum rate of cell death). (**i**) Western blots displaying total and phosphorylated protein levels of Erk1/2, Akt, p38MAPK and I*κ*B*α* in naïve cells (0) or upon TRAIL treatment. Depicted images correspond to one representative experiment out of at least three independent biological replicates. *α*-Tubulin, loading control. (**j**) Percentage of cells with high levels of cleaved PARP in populations treated with U0126 (MEK1/2 inhibitor), LY294002 (PI3 kinase inhibitor), PD 169316 (p38 MAP kinase inhibitor), Bay-11-7082 (NF-*κ*B inhibitor) or vehicle (DMSO) and either left unchallenged (control) or treated with TRAIL (5 h). (**k**) Percentage of cells with high levels of cleaved PARP in populations either pretreated with pan-caspase inhibitor (zVAD.fmk) or vehicle (DMSO) and then either left untreated (control) or challenged for 6 h with TRAIL. (**g**, **h**, **j** and **k**) Mean±standard deviation (S.D.) from three independent biological replicates is shown. ***P-value <0.0005, **P-value <0.005, *P-value <0.05. (**l**) Western blots displaying total and phosphorylated protein levels of Erk1/2, Akt, p38 and I*κ*B*α* in naïve cells (0) or upon TRAIL treatment in cells either pretreated with caspase-8 inhibitor Z-IETD-FMK, pan-caspase inhibitor zVAD.fmk or vehicle (DMSO). Depicted images correspond to one representative experiment out of at least three independent biological replicates. *α*-Tubulin, loading control

To study the molecular mechanisms underlying fractional survival in response to TRAIL treatment, we used isogenic populations of cancer cells derived from stepwise tumorigenesis models, in which normal cells are transformed into tumorigenic ones by introducing defined genetic elements.^34, 35^ These systems recapitulate the basics of TRAIL-induced tumor-selective apoptosis, as normal cells are resistant whereas tumorigenic cells show fractional survival.^12, 36^ Flow cytometry assays showed that fibroblast-derived stepwise tumorigenic cells (BJELR) challenged with high doses of TRAIL (1 *μ*g/ml; IC_50_ 30 ng/ml)^12^ displayed positive labeling for cleavage of initiator caspase-8 and PARP (poly(ADP-ribose)polymerase); yet, two subpopulations with 'low' or 'high' levels of these apoptosis-related markers were observed (Figures 1a and b; '1' and '2', respectively). This was validated in nine/nine independent sub-clonal populations, supporting that this response did not arise from clonal variations (Supplementary Figure S1a). To evaluate whether 'high' and 'low' levels of these markers correlated with death or survival in response to treatment, cells were exposed to TRAIL for 16 h. At this time point, the complete subpopulation of apoptotic cells was detached allowing for its direct collection, while surviving cells were collected after treatment with trypsin. Notably, high levels of cleaved PARP correlated with apoptotic cells, whereas lower mean intensity levels were observed for the subpopulation of surviving cells (Figures 1c–f) and, in line with our previous study,^12^ approximately 50% cells survived TRAIL challenge (Figures 1g and h). Besides inducing cell death, TRAIL also promoted Erk1/2, p38, Akt and IκB*α* phosphorylation at early time points, indicating that activation of non-apoptotic cascades occurs within the initial cell population (Figure 1i and Supplementary Figures S1b and c). Notably, pre-treatment of cells either with MEK1/2, PI3 kinase or p38 MAP kinase inhibitors prior to TRAIL challenge increased apoptosis. In contrast, inhibiting NF-*κ*B cascades by blocking IKK activity did not modify the apoptotic response to this ligand (Figure 1j and Supplementary Figure S1d). Collectively, these results indicate that activation of NF-*κ*B signaling does not play a key role in cell survival following TRAIL treatment, whereas functional Erk1/2, p38 and Akt kinase cascades are required to evade apoptosis in our model. Finally, incubation with zVAD.fmk pan-caspase inhibitor prior to TRAIL treatment blocked cell death (Figure 1k), supporting the notion that TRAIL triggers a caspase-dependent cell death program. On the other hand, pre-incubation with zVAD.fmk reduced Erk1/2 phosphorylation in response to TRAIL challenge but not that of p38 or Akt, and increased IκBα phosphorylation in response to treatment (Figure 1l). Similar results were observed when caspase-8 was selectively inhibited with z.IETD.fmk prior to TRAIL challenge (Figure 1l). Altogether, these results suggest that the activity of initiator procaspase(s) represents a first node of divergence for the activation of individual TRAIL-induced non-apoptotic pathways.

### TRAIL receptors form pro-apoptotic and pro-survival complex(es) in response to ligand binding

**Figure 2:**
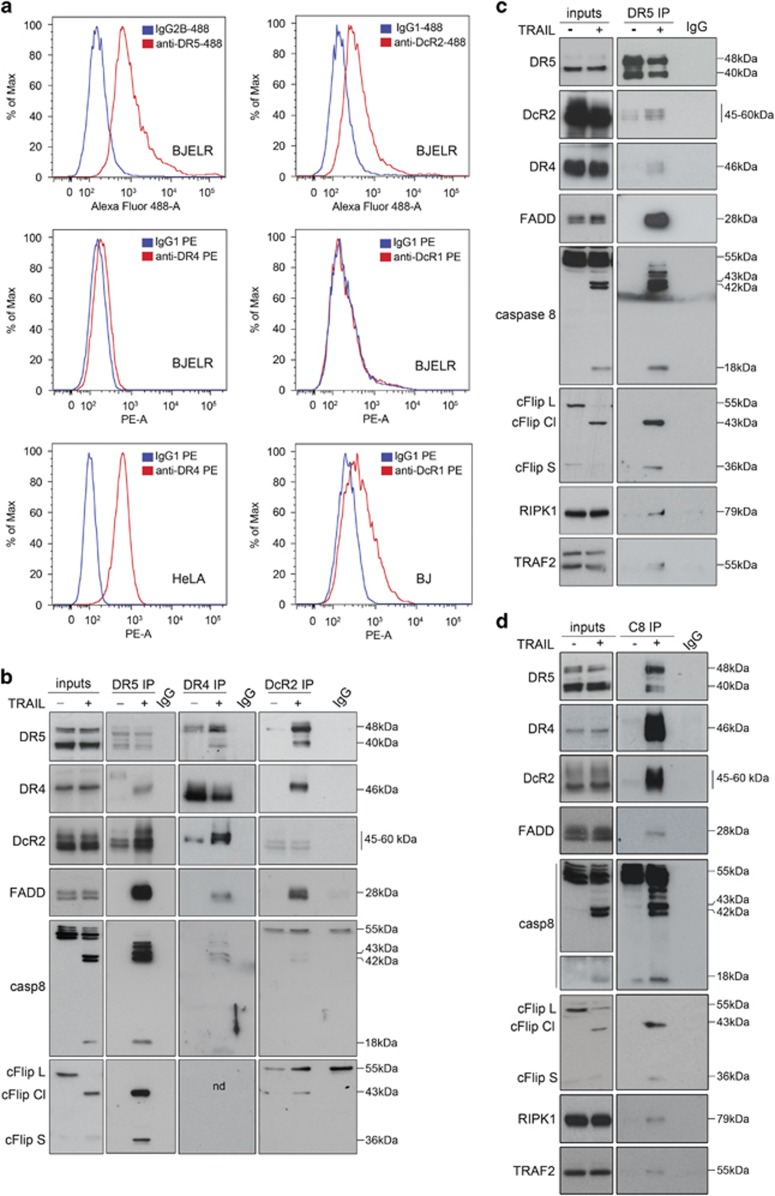
TRAIL induces the assembly of apoptotic and non-apoptotic signaling complex(es). (**a**) Surface levels of Death Receptor 5 (anti-DR5-488), Death Receptor 4 (anti-DR4 PE), Decoy Receptor 1 (anti-DcR1 PE) and Decoy Receptor 2 (anti-DcR2-488) in BJELR cells. Surface levels of Death Receptor 4 in Hela cells and surface level of Decoy Receptor 1 in BJ cells were used as a positive control for DR4 and DcR1 labeling, respectively. Isotypic IgG1PE or Alexa 488 or IgG2B Alexa 488 labeling was used as a control for background fluorescence. (**b**) Western blots assessing the co-immunoprecipitation of indicated proteins (canonical DISC components) with Death Receptor 5 (immunoprecipitation target; DR5 IP), Death Receptor 4 (immunoprecipitation target; DR4 IP) or Decoy Receptor 2 (immunoprecipitation target; DcR2 IP) from whole-cell lysates obtained from naïve cells (−) or cells treated with TRAIL for 30 min (+). NF, not detected. (**c**) Western blots assessing the co-immunoprecipitation of RIPK1 and TRAF2 as well as canonical DISC components (internal control) with Death Receptor 5 (DR5 IP) from whole-cell lysates obtained from naïve cells (−) or cells treated with 1 *μ*g/ml TRAIL for 30 min (+). (**d**) Western blots assessing the co-immunoprecipitation of RIPK1 and TRAF2 as well as canonical DISC components (internal control) with caspase-8 (immunoprecipitation target; C8 IP) from whole-cell lysates obtained from naïve cells (−) or cells treated with TRAIL for 30 min (+). (**b**–**d**) Depicted images correspond to one representative experiment out of three independent biological replicates. Immunoprecipitation using isotypic IgG1 (IgG) was used as a background control

Our results indicate that TRAIL triggers both apoptosis and non-apoptotic kinase signaling; yet the composition of TRAIL-induced signaling complex(es) and the individual roles of its components in the activation of non-apoptotic cascades remained an open question. To further elucidate the molecular basis underlying fractional survival, we set out to identify the molecular platforms assembled in response to TRAIL. Direct immunolabelling of TRAIL receptors followed by flow cytometry showed that DR4, DR5 and DcR2 were expressed at the cell surface, whereas no surface expression of DcR1 was detected (Figure 2a). Moreover, immunoprecipitation assays (IP) using whole-cell lysates and antibodies targeting either DR4, DR5 or DcR2 indicated that these receptors form heterocomplexes, which in turn recruit FADD, cFlip and caspase-8 (Figure 2b). Interestingly, DR5 IP following TRAIL challenge showed that RIPK1 and TRAF2 were both recruited to TRAIL-induced signaling complex(es) (Figure 2c). Furthermore, caspase-8 IP with whole-cell lysates confirmed that TRAIL induces the association of TRAIL receptors, RIPK1, TRAF2 and FADD with this initiator caspase (Figure 2d). Altogether, these results indicate that both canonical DISC members (FADD, caspase-8, cFlip) and proteins previously described to constitute a TRAIL-induced 'receptor depleted-secondary complex' (RIPK1 and TRAF2) are recruited to TRAILRs in response to this ligand.

**Figure 3:**
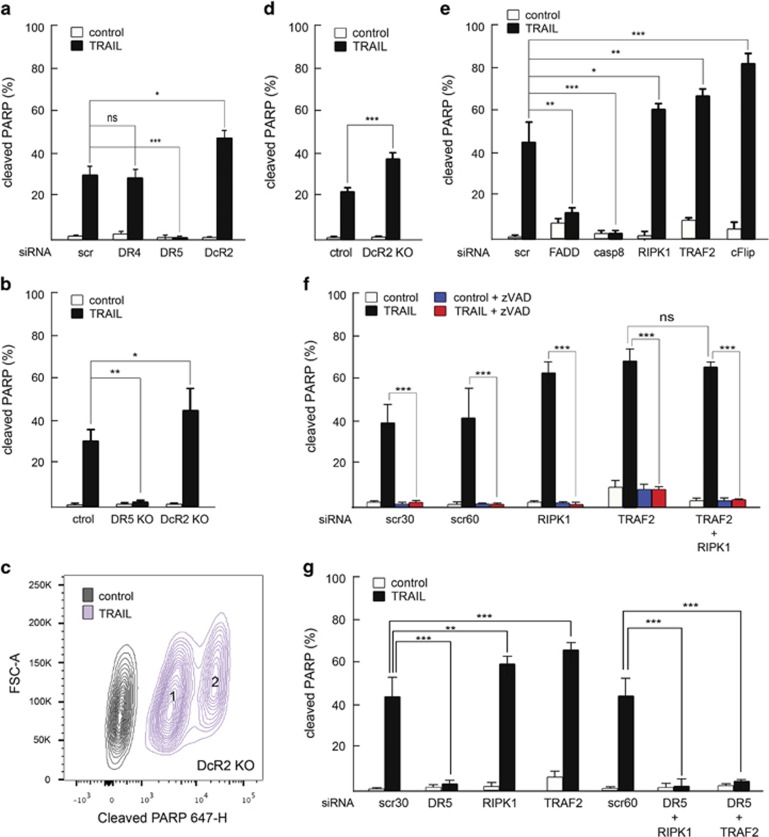
Role of TRAIL receptors and TRAIL-receptor-binding proteins in the triggering of apoptosis. (**a**) Percentage of cells with high levels of cleaved PARP in populations transfected with pooled siRNAs targeting *DR4* (DR4), *DR5* (DR5), *DcR2* (DcR2) mRNAs or non-targeting scramble siRNAs (scr) further left either untreated (control) or challenged with TRAIL (6 h). (**b**) Percentage of cells with high levels of cleaved PARP in naïve (control) or TRAIL-challenged (6 h) DR5 knockout (DR5 KO) or DcR2 knockout (DcR2 KO) cells as compared with controls (ctrol). (**c**) Contour plot for cleaved PARP immunolabelling in DcR2 knockout (DcR2 KO) cells either unchallenged (control) or treated 6 h with TRAIL (TRAIL). Subpopulations displaying low (survivors, '1') and high (apoptotic, '2') levels of PARP cleavage in treated samples are depicted. Image shows one representative experiment out of three independent biological replicates. (**d**) Percentage of cells with high levels of cleaved PARP in control and DcR2 KO naïve cells (control) or control and DcR2 KO cells challenged with TRAIL for 16 h (TRAIL). (**e**) Percentage of cells with high levels of cleaved PARP in cells transfected with siRNAs targeting *FADD* (FADD)*, caspase-8* (casp-8), *RIPK1* (RIPK1), *TRAF2* (TRAF2) or *cFlip* (cFlip) mRNAs or non-targeting scramble siRNAs (scr) further left either untreated (control) or challenged for 6 h with TRAIL (TRAIL). (**f**) Percentage of cells with high levels of cleaved PARP in populations transfected with siRNAs targeting *RIPK1* (RIPK1)*, TRAF2* (TRAF2) or *RIPK1* and *TRAF2* (RIPK1+TRAF2) *mRNAs* or non-targeting scramble siRNAs at concentrations corresponding to single (30 nM; scr 30) or double (60 nM; scr 60) transfections, further left either untreated (control) or challenged during 6 h with TRAIL (TRAIL). Results obtained in conditions of pan-caspase inhibition (control+ zVAD and TRAIL+zVAD) as compared with basal conditions of cell response (control and TRAIL) are shown. (**g**) Percentage of cells with high levels of cleaved PARP in cells transfected with siRNAs targeting *RIPK1* (RIPK1)*, TRAF2* (TRAF2)*, DR5* (DR5), *RIPK1* and *DR5* (RIPK1+DR5), *TRAF2* and *DR5* (TRAF2+DR5) mRNAs or non-targeting scramble siRNAs ('scr 30' and 'scr 60') further left either untreated (control) or challenged 6 h with TRAIL (TRAIL). (**a**, **b** and **d**–**g**) Mean±standard deviation (S.D.) from three independent biological replicates is shown. ***P-value <0.0005, **P-value <0.005, *P-value <0.05. NS, not significant

To analyze the role of each TRAIL receptor and associated proteins, post-transcriptional interference experiments followed by TRAIL treatment were performed and the rate of apoptosis was assessed (Figure 3a and Supplementary Figure S2). DR4 depletion did not affect the apoptotic rate in response to this ligand. In contrast, DR5 downregulation resulted in a major block of cell death, whereas the decrease of DcR2 levels enhanced apoptosis. In line with these results, a complete block in TRAIL-induced apoptosis was observed in DR5 knockout cells (Figure 3b and Supplementary Figure S3a). Interestingly, even though higher rates of cell death were observed in DcR2-deficient cells compared with controls (Figure 3b and Supplementary Figure S3a), still two populations displaying low and high levels of cleaved PARP were detected (Figure 3c) and a substantial fraction of the cellular population survived treatment (Figure 3d). Collectively these results reveal that both DcR2-dependent and DcR2-independent mechanisms counterbalance cell death and supports that DR5 is indeed the key receptor mediating TRAIL-induced cell death in this cellular system.

Regarding the proteins assembled to TRAIL receptors, we observed that FADD and caspase-8 are the essential adaptor protein and initiator caspase, as their downregulation blocked TRAIL-induced apoptosis. In contrast, depletion of cFlip, RIPK1 and TRAF2 increased cell death, highlighting their anti-apoptotic function (Figure 3e and Supplementary Figure S3b). Notably, based on its E3 ligase activity, TRAF2 promotes RIPK1-K63 ubiquitination enabling its anti-apoptotic function, whereas de-ubiquitination switches RIPK1 function to death promotion.^37, 38, 39, 40, 41^ To evaluate whether increased apoptosis under conditions of TRAF2 depletion relied on the same mechanistic principles as in wild-type cells, single RIPK1, TRAF2 and double RIPK1-TRAF2 knock-downs were performed (Supplementary Figure S3c) followed by TRAIL treatment. A similar sensitization to TRAIL-induced cell death was observed upon TRAF2 depletion as compared with TRAF2-RIPK1 double knock-down, excluding that the increased apoptotic response observed upon TRAF2 downregulation results from a switch of RIPK1 function from survival to death promotion (Figure 3f, compare TRAF2 TRAIL with TRAF2+RIPK1 TRAIL). Moreover, a complete block of apoptosis was observed by pre-incubation of TRAF2, RIPK1 and TRAF2-RIPK1-depleted cells with pan-caspase inhibitor zVAD.fmk (Figure 3f, compare (RIPK1, TRAF2, TRAF2+RIPK1) TRAIL with (RIPK1, TRAF2, TRAF2+RIPK1) TRAIL+zVAD), as well as upon TRAF2-DR5 and RIPK1-DR5 double knock-down (Figure 3g and Supplementary Figure S3d), further supporting that similar mechanistic principles underlie TRAIL-induced apoptosis in wild type, TRAF2 and RIPK1-depleted cells.

### Role of TRAIL-receptor complex(es) in the activation of survival cascades

**Figure 4:**
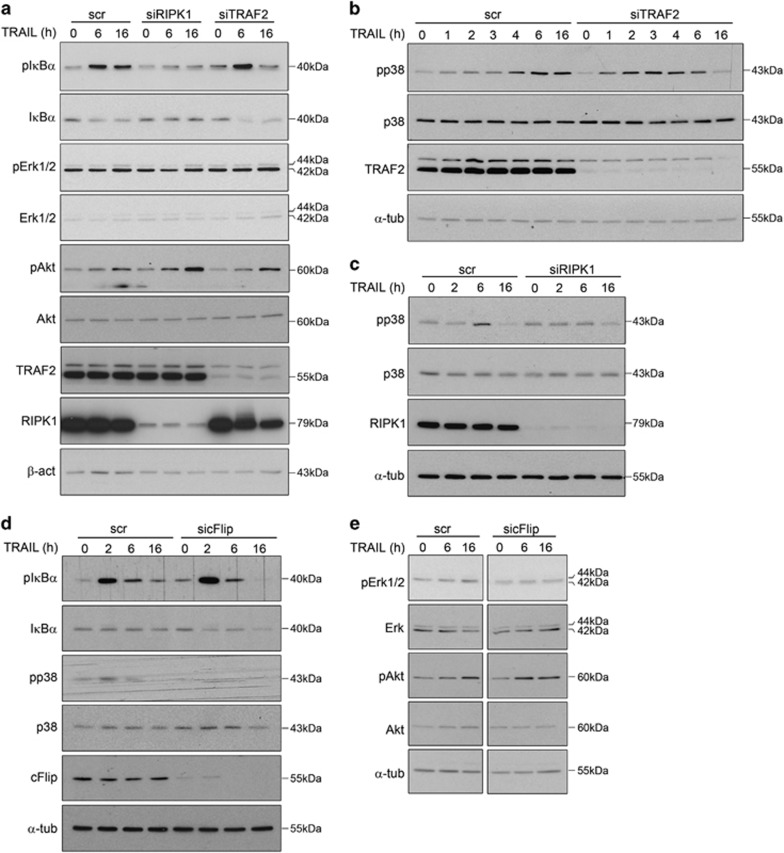

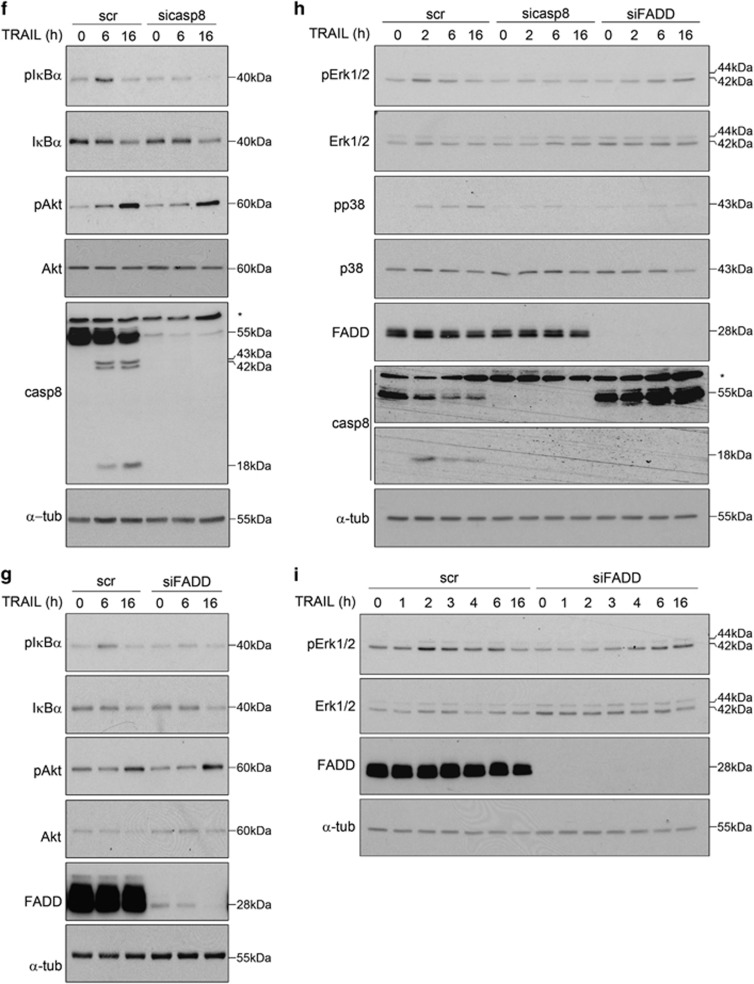
Role of TRAILRs-binding proteins in the triggering of non-apoptotic and pro-survival pathways. (**a**) Western blots displaying total and phosphorylated protein levels of Erk1/2, Akt and I*κ*B*α* in cells transfected with siRNAs targeting *RIPK1* (siRIPK1),*TRAF2* (siTRAF2) mRNAs or non-targeting scramble siRNAs (scr) further left either untreated (0) or challenged with TRAIL. (**b**) Western blots displaying total and phosphorylated protein levels of p38 in BJELR cells transfected with siRNAs targeting *TRAF2* (siTRAF2) mRNA or non-targeting scramble siRNAs (scr) further left either untreated (0) or challenged with TRAIL. (**c**) Western blots displaying total and phosphorylated protein levels of p38 in cells transfected with siRNAs targeting *RIPK1* (siRIPK1) mRNA or non-targeting scramble siRNAs (scr) further left either untreated (0) or challenged with TRAIL. (**d** and **e**) Western blots displaying total and phosphorylated protein levels of p38 and I*κ*B*α* (**d**), Erk1/2 and Akt (**e**) in cells transfected with siRNAs targeting *cFlip* (sicFlip) mRNA or non-targeting scramble siRNAs (scr) further left either untreated (0) or challenged with TRAIL. Uncropped image for (**e**) is depicted as Supplementary Figure S4a. (**f**) Western blots displaying total and phosphorylated protein levels of I*κ*B*α* and Akt in cells transfected with siRNAs targeting *caspase-8* (sicasp8) mRNA or non-targeting scramble siRNAs (scr) further left either untreated (0) or challenged with TRAIL. (**g**) Western blots displaying total and phosphorylated protein levels of I*κ*B*α* and Akt in cells transfected with siRNAs targeting *FADD* (siFADD) mRNA or non-targeting scramble siRNAs (scr) further left either untreated (0) or challenged with TRAIL. (**h**) Western blots displaying total and phosphorylated protein levels of Erk1/2 and p38 in BJELR cells transfected with siRNAs targeting *caspase-8* (sicasp8), *FADD* (siFADD) mRNAs or non-targeting scramble siRNAs (scr) further left either untreated (0) or challenged with TRAIL. (**i**) Western blots displaying total and phosphorylated protein levels of Erk1/2 in BJELR cells transfected with siRNAs targeting *FADD* (siFADD) mRNA or non-targeting scramble siRNAs (scr) further left either untreated (0) or challenged for the indicated time points with TRAIL. (**a**–**i**) Depicted images correspond to one representative experiment out of three independent biological replicates. *α*-Tubulin, loading control

We hypothesized that two different mechanisms could conceptually account for the modulation of apoptosis in response to TRAIL: (i) impeding the assembly of an apoptosis-proficient DISC and/or (ii) supporting the activation of pro-survival pathways. As our results demonstrate that Erk1/2, p38 and Akt cascades support cell survival in response to treatment (Figure 1j and Supplementary Figure S1d), we next evaluated the involvement of components of the TRAIL-induced signaling complex(es) (Figure 2c) in the activation of such non-apoptotic cascades. For that, levels of individual members of the complex(es) were knocked down and cells were treated with TRAIL. No major changes in TRAIL-induced Erk1/2, p38, Akt and IκBα phosphorylation were observed upon TRAF2 depletion compared with controls (Figures 4a and b), suggesting that TRAF2 anti-apoptotic function does not rely on its direct involvement in TRAIL-induced pro-survival signaling but it may be rather linked to its ability to ubiquitinate activated caspase-8 for degradation.^42^ In contrast, cFlip depletion hampered Erk1/2 and p38 phosphorylation (Figures 4d and e and Supplementary Figure S4), whereas RIPK1 was required for IκBα (Figure 4a) and p38 phosphorylation (Figure 4c) in response to TRAIL. These results support that RIPK1 and cFlip exert anti-apoptotic functions by – at least partially – modulating pro-survival signaling and reveal an opposite role of RIPK1 and TRAF2 in the TRAIL-induced activation of inflammatory cascades. Indeed, depletion of RIPK1 diminished the production of pro-inflammatory cytokines in response to TRAIL, whereas an increase in mRNA levels of pro-inflammatory proteins in response to this ligand was detected in conditions of TRAF2 downregulation (Supplementary Figure S5a).

**Figure 5:**
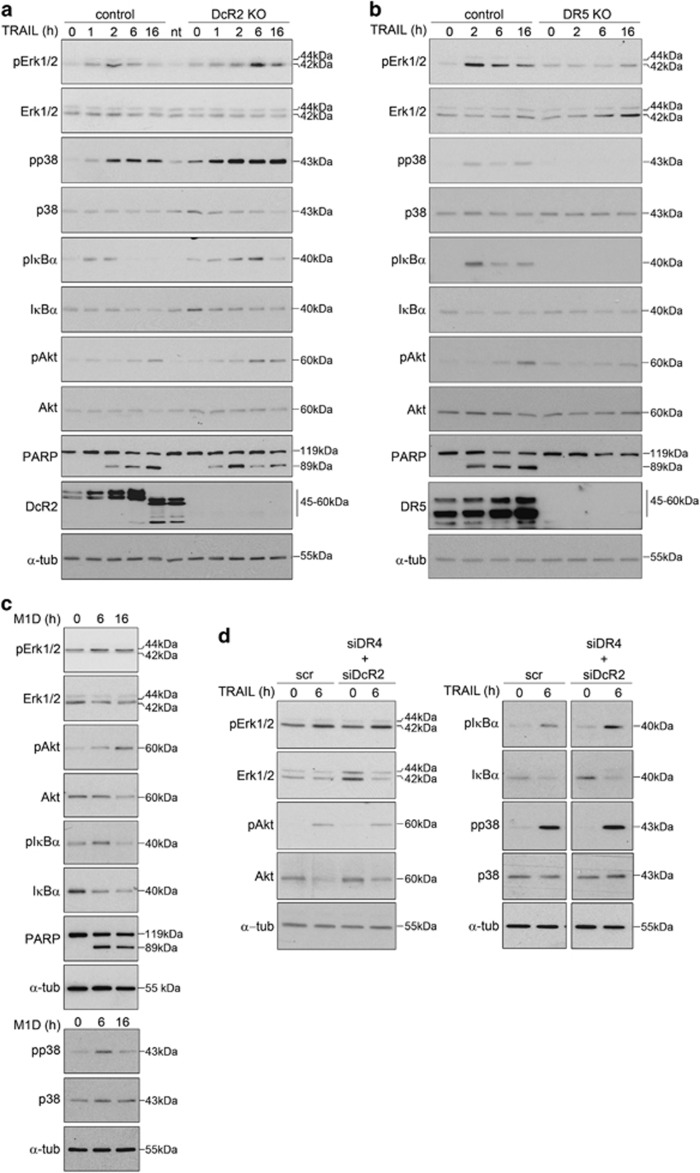
Role of TRAIL receptors in the triggering of non-apoptotic and pro-survival pathways. (**a** and **b**) Western blots displaying total and phosphorylated protein levels of Erk1/2, Akt, p38 and I*κ*B*α* in clonal populations of DcR2 knockout (DcR2 KO, **a**), DR5 knockout (DR5 KO, **b**) and control BJELR cells in basal conditions (0) or after treatment with TRAIL for the indicated time points. PARP cleavage is shown as a control for TRAIL treatment whereas DcR2 (**a**) or DR5 (**b**) is depicted as knockout controls. Images correspond to one representative experiment out of two independent biological replicates. (**c**) Western blots displaying total and phosphorylated protein levels of Erk1/2, Akt, p38 and I*κ*B*α* in BJELR cells challenged with DR5-selective TRAIL-mimetic peptide M1D (10 *μ*M) as compared with controls (0). (**d**) Western blots displaying total protein and phosphorylation levels of Erk1/2, Akt, p38 and I*κ*B*α* in BJELR cells transfected either with siRNAs targeting *DR4* and *DcR2* (siDR4+siDcR2) mRNAs or non-targeting scramble siRNAs (scr) further left untreated (0) or challenged for 6 h with TRAIL. Uncropped image is shown as Supplementary Figure S4b. Images correspond to one representative experiment out of at least two independent biological replicates. *α*-Tubulin, loading control

Given that FADD and caspase-8 are reported key components of the called 'secondary complex' supporting survival in response to members of TNF*α* family of cytokines,^23^ the role of these two proteins was also analyzed. We found that, besides being key components for TRAIL-induced cell death (Figure 3e and Supplementary Figure S3b), they were also required for TRAIL-induced IκB*α* and p38 phosphorylation (Figures 4f–h). Moreover, Erk1/2 phosphorylation was blocked in conditions of caspase-8 depletion, whereas a delayed response was observed upon FADD knock-down (Figures 4h and i). Finally, the TRAIL-dependent increase of Akt phosphorylation was seen in all cases when individual members of the TRAIL-induced signaling complex(es) were depleted (Figures 4a, e, f and g), indicating that as yet unidentified signaling platforms are involved in activating this particular kinase cascade. In that regard, recent reports support the notion that DcR2 modulates the activation of Akt signaling by TRAIL.^13^ However, knock out of DcR2 did neither affect TRAIL-induced Akt phosphorylation nor that of Erk1/2, p38 or IκBα, or the induction of pro-inflammatory cytokines (Figure 5a and Supplementary Figure S5b). Collectively, these results indicate that DcR2 tunes TRAIL-induced apoptosis by interference with the required proximity between zymogens at the DISC,^43, 44, 45^ thus hampering the formation of an apoptosis-proficient DISC rather than by modulating TRAIL-induced survival and/or inflammatory signaling.

As DcR2 depletion did not affect TRAIL-induced kinase phosphorylation (Figure 5a) and considering that DR4 was not key in the response to TRAIL (Figure 3a and Supplementary Figure S2c vs d), we analyzed the role of DR5 in the activation of non-apoptotic cascades. Phosphorylation of Erk1/2, p38, Akt and IκBα was blocked by DR5 depletion (Figure 5b). Moreover, treatment with TRAIL-mimetic peptides selectively targeting DR5^46^ promoted Erk1/2, Akt, p38 and IκBα phosphorylation (Figure 5c) and similar phosphorylation patterns as in controls were observed in DR4-DcR2 double knock-down cells in response to TRAIL (Figure 5d and Supplementary Figures S6a and b). Interestingly, selective activation of DR5 induced both cell death and activation of non-apoptotic pathways in two other human cancer cell lines which express high levels of DR4 on their surface (Supplementary Figure S7). Altogether, these results strongly suggest that besides acting as a main apoptotic inducer (Figure 3a and Supplementary Figure S2c vs e) DR5 mediates also the TRAIL-dependent activation of non-apoptotic signaling, independently of DR4 or DcR2.

### Pro-survival and pro-apoptotic TRAIL-signaling complex(es) are assembled via DR5 at the plasma membrane

**Figure 6:**
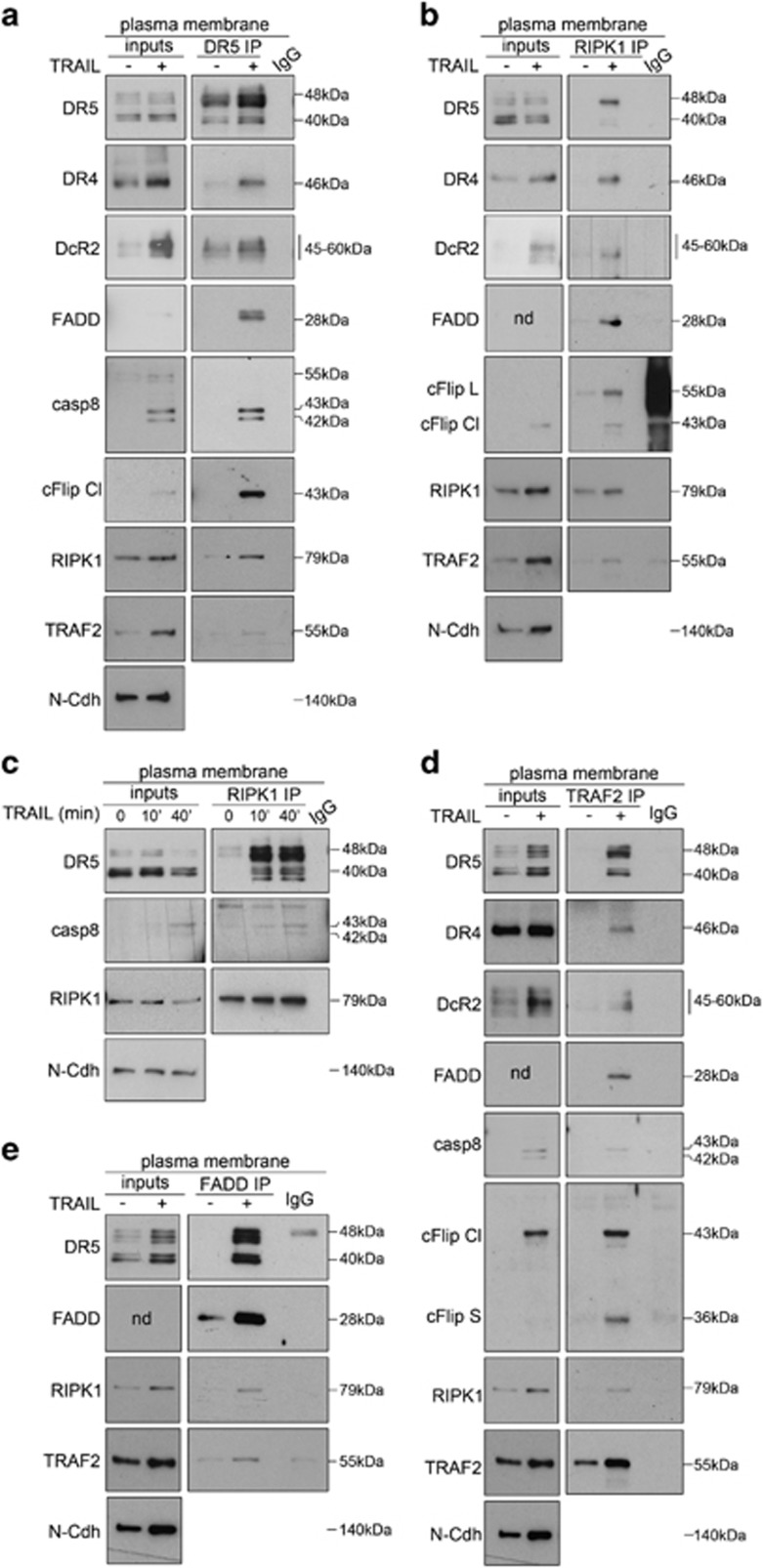
Apoptotic and pro-survival TRAIL-induced signaling complex(es) are assembled at the plasma membrane. (**a**) Western blots assessing the co-immunoprecipitation of canonical DISC components (DR4, DcR2, FADD, caspase-8, cFlip) as well as RIPK1 and TRAF2 with Death Receptor 5 (DR5 immunoprecipitation: DR5 IP). (**b** and **c**) Western blots assessing the co-immunoprecipitation of DR5, DR4, DcR2, FADD, cFlip and TRAF2 (**b**) as well as caspase-8 (**c**) with RIPK1 (RIPK1 immunoprecipitation: RIPK1 IP). (**d**) Western blots assessing the co-immunoprecipitation of canonical DISC components (DR4, DR5, DcR2, FADD, caspase-8, cFlip) as well as RIPK1 with TRAF2 (TRAF2 immunoprecipitation: TRAF2 IP). (**e**) Western blots assessing the co-immunoprecipitation of DR5, RIPK1 and TRAF2 with FADD (FADD immunoprecipitation: FADD IP). (**a–e**) Immunoprecipitations were performed from the purified plasma membrane (plasma membrane) obtained from either naïve cells (−) or cells treated with TRAIL (30 min '+' unless indicated otherwise in **c**). Immunoprecipitation using isotypic IgG1 (IgG) was used as a background control. Protein levels of indicated proteins at the plasma membrane are depicted as 'inputs'. Same blots are depicted as inputs for (**d**) and (**e**) as TRAF2 and FADD immunoprecipitation experiments were performed in parallel using the same protein input. Depicted images correspond to one representative experiment out of at least two independent biological replicates. N-Cadherin (N-Cdh) is shown as a plasma membrane marker. Controls for plasma membrane purification are depicted as Supplementary Figure S5. ND, not detected

**Figure 7:**
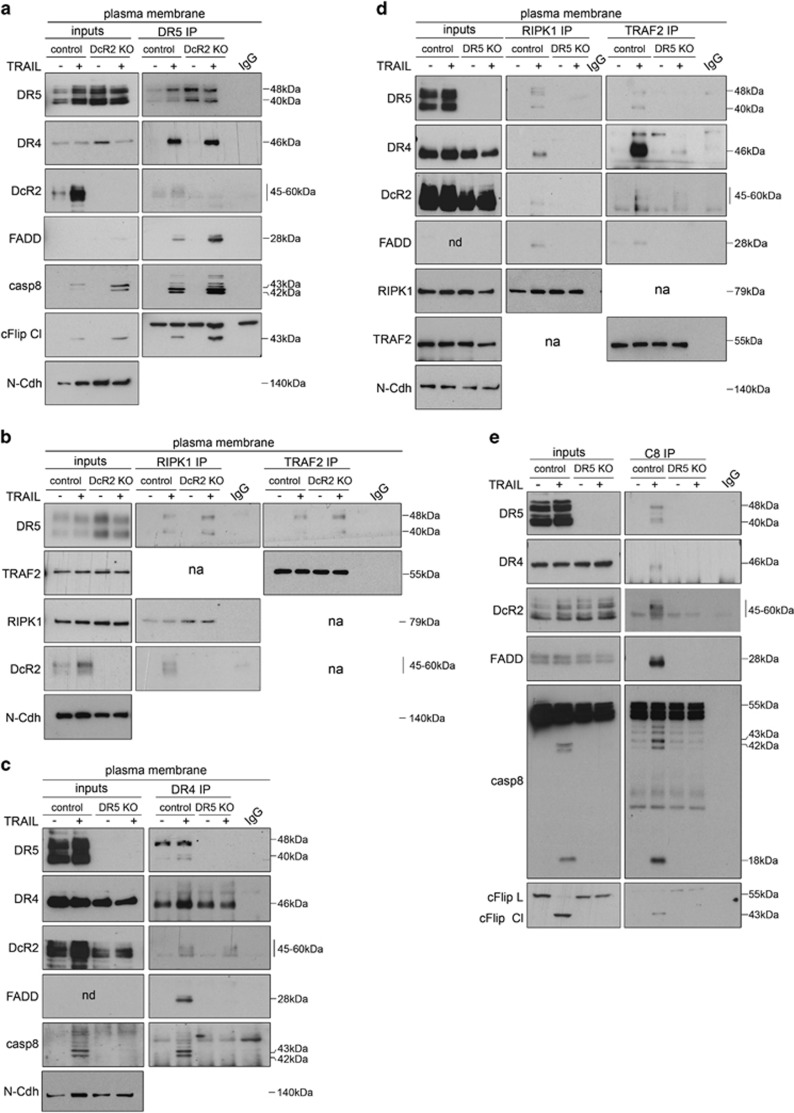
Role of TRAIL receptors in the formation of TRAIL-induced signaling platforms. (**a**) Western blots assessing the co-immunoprecipitation of canonical DISC components (DR4, DcR2, FADD, caspase-8, cFlip) with Death receptor 5 (DR5 immunoprecipitation: DR5 IP) in plasma membrane fractions obtained from DcR2 knockout (DcR2 KO) and control BJELR cells either left untreated (−) or challenged with TRAIL (30 min '+'). (**b**) Western blots assessing the co-immunoprecipitation of DR5 with RIPK1 (RIPK1 immunoprecipitation: RIPK1 IP) or TRAF2 (TRAF2 immunoprecipitation: TRAF2 IP) in DcR2 knockout (DcR2 KO) and control BJELR cells either left untreated (−) or challenged with TRAIL (30 min '+'). (**c**) Western blots assessing the co-immunoprecipitation of canonical DISC components (DR5, DcR2, FADD, caspase-8) with Death receptor 4 (DR4 immunoprecipitation: DR4 IP) in plasma membrane fractions obtained from DR5 knockout (DR5 KO) and control BJELR cells either left untreated (−) or challenged with TRAIL (30 min '+'). (**d**) Western blots assessing the co-immunoprecipitation of canonical DISC components (DR5, DR4, DcR2, FADD) with RIPK1 (RIPK1 immunoprecipitation: RIPK1 IP) or TRAF2 (TRAF2 immunoprecipitation: TRAF2 IP) in DR5 knockout (DR5 KO) and control BJELR cells either left untreated (−) or challenged with TRAIL (30 min '+'). (**e**) Western blots assessing the co-immunoprecipitation of canonical DISC components (DR5, DR4, DcR2, FADD, cFlip) with caspase-8 (caspase-8 immunoprecipitation: C8 IP) in whole-cell lysates from DR5 knockout (DR5 KO) and control BJELR cells either left untreated (−) or challenged with TRAIL (30 min '+'). (**a**–**c** and **e**) Controls for plasma membrane purification are depicted as Supplementary Figure S5. ND, not detected; NA, not analyzed

While the model proposing that formation of RIPK1 and TRAF2-containing TRAIL-induced 'secondary complex' supports cell survival after exposure to TRAIL is accepted in the field,^23^ the role of individual TRAIL receptors and subcellular compartment(s) where such a complex is assembled remains elusive. To shed light on this, we first studied whether all proteins assemble during the initial steps of TRAIL signaling. Cells were treated with TRAIL, the plasma membrane fraction was purified (Supplementary Figure S8a) and the composition of TRAIL-induced complex(es) was assessed by co-immunoprecipitation experiments using antibodies directed against DR5 (Figure 6a and Supplementary Figure S8a), RIPK1 (Figures 6b and c and Supplementary Figures S8b and c and S9), TRAF2 (Figure 6d and Supplementary Figure S8d) or FADD (Figure 6e and Supplementary Figure S8d). Results showed that FADD, caspase-8, cFlip, RIPK1 and TRAF2 were recruited to TRAIL-receptor heterocomplexes (DR4–DR5–DcR2) (Figures 6a–e), yet the role of individual receptors for the assembly and final composition of these platform(s) remained unclear. As DcR2 depletion increased the rate of cell death in response to TRAIL (Figures 3a and b and Supplementary Figure S2c vs f), we analyzed how this decoy receptor modulates the composition of TRAIL-signaling complex(es). Increased co-immunoprecipitation of FADD and caspase-8 with DR5 was observed in DcR2 knockout cells as compared with controls (Figure 7a and Supplementary Figure S8e), whereas co-immunoprecipitation of TRAF2 and RIPK1 with DR5 was not altered (Figure 7b and Supplementary Figure S8f). These results indicate that DcR2 tunes the final apoptosis outcome at initial steps of the response by preventing the recruitment and proximity-induced activation of initiator caspase-8 without having a major impact on TRAF2 or RIPK1 assembly to DR5.

Recapitulating, our results indicate that despite being assembled into TRAIL-induced complexes, DR4 does not play a key role in TRAIL signaling in our model, whereas DcR2 balances cell death mainly by regulating caspase-8 activation. Unexpectedly, DR5 was identified as the key receptor mediating activation of apoptosis and non-apoptotic signaling in response to TRAIL. Thus, we hypothesized that DR5 mediates assembly of both TRAIL-induced pro-apoptotic and/or pro-survival signaling complex(es) independently of other TRAIL receptors. Indeed, even though weak co-immunoprecipitation of DR4 and DcR2 was observed in DR5-depleted cells in response to TRAIL (Figure 7c and Supplementary Figure S8g), TRAF2 recruitment was severely downregulated (Figure 7d and Supplementary Figure S8h) and no significant co-immunoprecipitation of FADD, cFlip, caspase-8 or RIPK1 with DR4 could be detected (Figures 7c–e and Supplementary Figure S8g and h). Giving that DR4 displays a wild-type death domain (Supplementary Figure S10) we hypothesize that the lack of recruitment of a proficient DISC may rely – at least partially – on the low plasma membrane level of this receptor in our cell model.

Altogether these data indicate that DR5 promotes the assembly of both pro-apoptotic and pro-survival proteins into membrane-proximal TRAIL-induced signaling complex(es) independently of DR4 and DcR2, thereby leading to fractional survival and TRAIL resistance in cancer cells.

## Discussion

Following the discovery of 'cancer cell-selective' apoptosis by TRAIL, several strategies have been explored for cancer therapy.^32, 33^ However, resistance and persistence of residual disease were the most prevalent scenarios, supporting the concept that the apoptotic potential of this pathway is in dynamic balance with opposing survival strategies.^47, 48^ Indeed, our study reveals that TRAIL triggers not only apoptosis but activates concomitantly non-apoptotic signaling through a single death receptor, supporting 'fractional survival'.^12, 28^ The isogenic nature of our experimental models excludes that non-apoptotic responses result from genetic heterogeneity of the cell population.^12^ Previously, a 'non-genetic' basis of death *versus* survival responses was attributed to stochastic differences in the levels and activities of pro and anti-apoptotic proteins.^29^ If such differences were the sole factor modulating cell fate, fractional survival should be observed also under continuous treatment, as stochastic differences should be maintained once the 'time-to-death' between daughter cells has completely diverged.^29^ However, sustained resistance to death was observed under persistent TRAIL challenge and only numerous days after treatment withdrawal, cells reverted to the fractional killing seen on first exposure,^12^ indicating that TRAIL-dependent processes support treatment-tolerance.

Notably, our data support that DR5 activation simultaneously induces pro-death and pro-survival signaling. In contrast to TNFRI system,^49^ both TRAIL-induced pro-survival and pro-apoptotic complex(es) were rapidly formed at the plasma membrane. Notably, our co-IP studies suggest that TRAIL induces the formation of dynamic composite platform(s) rather than separate pro-apoptotic and pro-survival entities. We propose that the location of these platforms within different plasma membrane domains directs signaling towards apoptosis or non-apoptotic pathways. Supporting this concept, in TRAIL-resistant cancer cells, binding of RIPK1 to DR5 maintained the assembled complexes outside of lipid rafts, thus preventing caspase-8 activation and promoting non-apoptotic signaling.^50^ In contrast, DR5 complexes assembled within lipid rafts lead to efficient caspase activation in TRAIL-sensitive cancer cells.^51^ Thus, in fractional survival (i) lipid raft-located and raft-evicted complexes may form in different cells or (ii) TRAIL-signaling platforms assemble within single cells concomitantly at both plasma membrane locations and ultimately the kinetics of formation and/or ratios between raft-located/raft-evicted complexes will define whether a cell dies or survives. Importantly, it remains to be clarified whether the plasma membrane location of such TRAIL-induced platforms is stochastic or orchestrated by precise molecular mechanisms. Considering that DR4 is crucial for cell death induction in certain cancer cells,^52, 53, 54^ it will be of utmost relevance to assess if this receptor also bears dual pro-death/pro-survival functions for evaluating the therapeutic strategies to be applied when both death receptors are significantly expressed at the plasma membrane of cancer cells.

Interestingly, it was reported that caspase-8 mutants with hampered activity promote the activation of pro-survival cascades.^55^ Here, we show that activation of inflammatory cascades occurs independently of caspase enzymatic function. Yet, in line with recent findings showing that procaspase-8 acts as a scaffold for recruitment of non-apoptotic proteins to activate inflammatory and proliferative signaling,^56, 57^ our experiments support that procaspase-8 is necessary for the activation of TRAIL-induced non-apoptotic pathways. Moreover, caspase inhibition stabilized recruitment of RIPK1 into TRAIL-induced complex(es) (data not shown). Therefore, we propose that TRAIL promotes the assembly of complexes composed of the same proteins but at different relative ratios and an excess of components blocking caspase activation (i.e. cFlip, TRAF2, DcR2) paves the way towards the assembly and/or stabilization of pro-survival platforms leading to resistance.

Because TRAIL is part of the natural cancer immunosurveillance system,^22^ shifting the response to this cytokine from apoptosis to pro-survival is likely a strategy of cancer cells to survive and proliferate *in vivo*. Therefore, the persistence of residual tumors following treatment should no longer be seen as an 'inability' of cells to respond but rather as 'adaptive resistance' in which cancer cells may actually profit from the therapeutic strategy for their own propagation. Notably, targeting death receptors with selective ligands has been considered as the best option to avoid sequestration of the ligand by decoy receptors that decreases caspase activation and, eventually, leads to activation of non-apoptotic cascades.^11, 13^ In that respect, we show that death receptor-selective targeting will not be enough to circumvent survival and/or metastasis-promoting functions of the TRAIL cascade, as a single death receptor suffices to feed into pro-death and pro-survival and migration networks. Indeed, we previously described the potent tumoricidal action of DR5-selective TRAIL-mimetic peptides (M1d) for xenografted colon cancer cells (HCT116). However, even though a prominent tumorcidal action was observed in M1d-treated animals, the persistence of residual tumors was the prevalent scenario at end time points of the experiments.^46^ Notably, we now demonstrate that M1d treatment also activates pro-survival/inflammatory cascades in HCT116 cells *in vitro*. Additional studies are required to validate the concomitant induction of pro-death/pro-survival signaling by DR5-selective targeting *in vivo* and whether sustained treatment with selective ligands could shift the tumoricidal effect towards tumor promotion.

Concluding, our results strongly disfavor the use of death receptor-selective agonists in monotherapies and argue for the need of new concepts emerging from basic research to develop novel types of combination therapies. Particularly, our data support the development of further *ex vivo* and *in vivo* experiments combining TRAIL-receptor agonists with compounds inhibiting TRAIL-induced pro-survival signals (e.g MAPK inhibitors) to evaluate the possibility of boosting TRAIL-induced cancer-selective death while inhibiting its tumor-promoting features.

## Materials and Methods

### Antibodies

Antibodies used for western blot were purchased from Cell Signaling (The Netherlands) (p38MAPK (cat. # 9212), p-p38MAPK (Thr180/Tyr182; cat. # 9211), DR5 (cat. # 3696), DcR2 (cat. # 8049), TRADD (cat. # 3684), p44/42MAPK (cat. # 9102), p-p44/42MAPK (Thr202/Tyr204; cat. # 9101), Akt (cat. # 9272), pAkt (Thr308; cat. # 4056), pI*κ*B*α* (Ser32/36; cat. #9246), I*κ*B*α* (cat. # 4814), PARP (cat. # 9542), NEMO (cat. # 2685), NEMO (cat. # 2695), caspase-8 (cat. # 9746), RIPK1 (cat. # 3493), TRAF2 (cat. # 4724), Anti-mouse IgG-HRP-linked antibody (cat. # 7076), Anti-rabbit IgG-HRP-linked antibody (cat. # 7074)); Santa Cruz Biotechnology (Dallas, TX, USA) (*β*-actin sc-1615, TRAF2 sc-7187, *α*-tubulin sc-32293); Millipore (France) (DR4 cat. AB16955, mouse anti-rabbit light chain-specific HRP-conjugated monoclonal antibody cat. MAB201P, FADD cat. # 06711); BD Pharmingen (San Jose, CA, USA) (FADD cat. # 556402, N-Cadherin cat. # 610920); AdipoGen (Switzerland) (cFlip AG-20B-0056-C100); and Abcam (Cambridge, MA, USA) (anti-EEA1 cat. AB2900). Antibodies for immunoprecipitation of DR5 (AF631), DR4 (AF347), DcR2 (AF633), TRAF2 (AF3277) and normal goat IgG (AB-108-C) were purchased from R&D Systems (Minneapolis, MN, USA). Antibodies for caspase-8 immunoprecipitation (sc-6136) were obtained from Santa Cruz Biotechnology; RIPK1 (cat. # 51-6559GR) and FADD (cat. # 556402) from BD Pharmingen; and normal mouse IgG was purchased from Jackson ImmunoResearch (West Grove, PA, USA) (015-000-003). For flow cytometry assays, cleaved caspase-8 (cat. # 9496), cleaved caspase-8 PE (cat. # 12602), cleaved PARP Alexa 647 (cat. # 6987) and cleaved PARP PE (cat. # 8978) were purchased from Cell Signaling, and anti-rabbit Alexa 488 (cat. # A21206) from Life Technologies (Carlsbad, CA, USA). For surface expression of TRAIL receptors, Mouse IgG1 (IC002G), Mouse IgG2B (IC0041G), DcR1 (FAB6302P), DcR2 (FAB633G) and DR5 (FAB6311G) are from R & D Systems; DR4 (804-297TD-T100) from Enzo Life Sciences (Switzerland); and DR4 (cat. # 854.852.010) and DR5 (cat.# 854.862.010) from Diaclone (France).

### Inhibitors

P38MAPK inhibitor PD 169316 was purchased from Calbiochem (San Diego, CA, USA) (cat. # 513030), MEK inhibitor U0126 from Promega (Switzerland) (cat. # V112A), PI3K inhibitor LY294002 from Cell Signaling (cat. # 9901), and inhibitor of I*κ*B*α* phosphorylation Bay-11-7082 from Sigma (Germany) (cat. # B5556). The final working concentrations of inhibitors were: PD 169316 25 *μ*M, U0126 20 *μ*M, LY294002 50 *μ*M, and Bay-11-7082 40 *μ*l. Cells were exposed to 1 h pre-treatment with indicated inhibitors prior to TRAIL treatment.

Pan-caspase inhibitor Z-VAD.fmk (cat. # FMK001) and caspase-8 inhibitor Z-IETD-FMK (cat. # FMK007) were purchased from R & D systems. Working concentrations for Z-VAD.fmk and Z-IETD-FMK were 100 *μ*M. PD 169316, U0126, LY294002, Bay-11-7082 and Z-VAD-FMK were reconstituted in DMSO. Equal volumes of vehicle were added to monitor any DMSO-related effects (indicated in figures as 'control').

### TRAIL

Purification of recombinant human TRAIL was performed as described by Kim *et al.* (2004).^58^ Briefly, *Escherichia coli BL21(DE3) were transformed with* 1 *μ*g pET9A plasmid (Novagen, Madison, WI, USA)-expressing TRAIL (aa 114-281). Next, 10 ml LB medium cultures containing selection antibiotics and 100 *μ*M zinc sulfate (ZnSO_4_) were inoculated with bacterial clones and grown at 18–21 °C and 190 r.p.m. Once the culture has reached an optical density (OD) of 0.3–0.6, the bacterial expression of the rhTRAIL gene was induced adding the lactose analog isopropyl *β*-d-1-thiogalactopyranoside (IPTG) to a final concentration of 0.5 mM. When the culture has reached an OD of 0.3–0.6, TRAIL expression was induced with 1 mM IPTG at 18–20 °C and 190 r.p.m. until the OD has doubled. Bacterial cultures were centrifuged at 6000  r.p.m. and 4 °C for 30 min. Bacteria were homogenized in lysis buffer (50 mM sodium phosphate, pH 8.0, 300 mM NaCl, 10 mM imidazole, and 10 mM *β*-mercaptoethanol or 5 mM dithiothreitol). Recombinant TRAILs were isolated from the soluble fraction using Ni-NTA agarose beads (Qiagen, The Netherlands, cat. # 30210) after washing with lysis buffer containing 20 mM imidazole. Isolated proteins were dialyzed against phosphate-buffered saline with 10 mM *β*-mercaptoethanol. The purity of the recombinant TRAIL proteins was confirmed by SDS-PAGE and Coomassie Blue gel staining. Characterization of the binding affinity and specificity of purified rhTRAIL to TRAIL receptors was described in Pavet *et al.* (2010).^46^

### Cell lines

BJELR cells from stepwise tumorigenesis system (BJ-derived model) were obtained from Dr. Hahn^34^ and cultured in DMEM (1 g/l glucose)+Medium 199 (4:1)+10% FCS heat inactivated. Clonal populations of BJELR cells were generated by single-cell cloning. For selection, medium of BJELR cells was freshly supplemented with 100 *μ*g/ml hygromicine (selection for hTERT expression), 400 *μ*g/ml G418 (selection for SV40 ER expression) and 0.5 *μ*g/ml puromycin (selection for HRas V12 expression). Expression of SV40 ER and *H-rasV12* was confirmed by western blot. Cells were grown at 37 °C, 5% carbon dioxide (CO_2_). Cells were plated at a confluency of 10^6^ cells in 10 cm Petri dishes (6010 mm^2^ surface) with 10 ml fresh medium. Cells were passed every 48 h and were kept in culture for a maximum of 15 passages. For treatment, 1 *μ*g/ml of TRAIL was added directly on the medium of adherent cells. Mycoplasma contamination was analyzed by PCR.

### Samples

For each experiment, independent cell cultures, treatment and processing of the material were conducted (stated as 'biological replicates'). The number of biological replicates for each experiment is indicated in figure legends.

### siRNA-mediated knock-down

ON-TARGET Plus siRNAs were purchased from Dharmacon (Lafayette, CO, USA). RIPK1 (L-0044445-00-0005), TRAF2 (L-005198-00-0005), FADD (L-003800-00-0005), DcR2 (J-008092-14 and J-008092-15), caspase-8 (J-003466-16), cFlip (L-003772-00-0005) were used. For DR4 knock-down a pool of two individual siRNA (J-008090-09-0005 and J-008090-08-0005) was used. DR5 knock-down was achieved utilizing pool of three single siRNA (J-004448-06-0005, J-004448-07-0005 and J-004448-08-0005). Transfect ion with non-targeting scrambled siRNA (D-001810-01-20) was used as a background control. Efficient knock-downs were achieved at 48 h post-transfection with 30 nM siRNAs. Transfection was done using RNAi Max Lipofectamine (Invitrogene cat. # 13778150). Transfection protocol for a surface area of 903 mm^2^ was as follows: Initially 4 *μ*l of desired siRNAs (20*μ*M stock) were diluted in 250 *μ*l Opti-MEM (Opti-MEM Reduced serum medium, GlutaMAX Supplemented, ThermoFisher Scientific (Germany), cat. # 51985034), mixed and incubated for 15 min at room temperature (Mix 1). In parallel, 11.25 *μ*l of RNAi Max Lipofectamine were diluted in 250 *μ*l Opti-MEM, mixed and incubated for 15 min at room temperature (Mix 2). Mix 1 and Mix 2 were combined, incubated at room temperature for 20 min and placed into the well, 5 min later 250 000 cells in 2 ml complete medium were added (final volume was 2.5 ml/well). Twenty-four hours post-transfection medium was renewed and cells were incubated for additional 24 h prior to collection or challenge with 1 *μ*g/ml TRAIL.

### CRISPR-Cas9 mediated knockout of DR5 and DcR2

Knockout cell lines were generated using double nickase plasmids purchased from Santa Cruz Biotechnology. BJELR cells were transfected with 200ng/ml of DR5 double nickase plasmid (sc-4010002-NIC), DcR2 double nickase plasmid (sc-403389-NIC) or control double nickase plasmid (sc-437281-NIC) using Lipofectamine LTX (Invitrogene cat. # 15338100). The transfection protocol for a surface area of 903 mm^2^ was as follows: BJELR cells were plated at 150 000/903 mm^2^. After 24 h, 180 *μ*l Opti-MEM were mixed with 3 *μ*l of Plus reagent (Invitrogene cat. # 15338100) and incubated for 15 min at room temperature. Double nickase plasmids were added to this solution, mixed and incubated for 20 min at room temperature (Mix 1). In parallel 7.2 *μ*l of Lipofectamine LTX was diluted in 180 *μ*l of Opti-MEM, mixed and incubated for 20 min at room temperature (Mix 2). Mix 1 and Mix 2 were combined, mixed, incubated at room temperature for 20 min and added to the medium of growing cells (direct transfection). Final volume was 2.5 ml per well.

Twenty-four hours post-transfection cells were trypsinized using 0.05% Trypsin-EDTA (ThermoFisher Scientific, cat. # 25300062). Single cells were collected in 96-wells plates by FACS. Twenty-four hours later, wells containing single cells were selected under the microscope and supplemented with additional 50 *μ*l of medium. Cell culture medium was then replaced (100 *μ*l) every 48 h. When clones reached ~80% confluency in 96-well plates, they were transferred into 24-wells plates. Each clone was analyzed by western blot for the presence or absence of DR5 or DcR2 protein. Cells transfected with control double nickase plasmid (sc-437281-NIC) were used as a control in all experiments involving DcR2 or DR5 KO cells.

### Apoptosis measurement

Apoptotic cell death was determined using flow cytometry by assessing the percentage of cells displaying positive staining for 'high levels' of cleaved PARP or cleaved caspase-8 (Figures 1a and b, population '2'). BJELR cells were plated at a density of 240 000 cells per 903 mm^2^; 24 h later cells were either left untreated or challenged with TRAIL (1 *μ*g/ml). Further, cells were collected by trypsinization and centrifuged. Pellet was re-suspended in 50 *μ*l of 1 × PBS and fixed with 1 ml of methanol (kept at −20 °C prior to use) for 5 min at room temperature. Next, methanol was discarded by centrifugation and cells were washed once with 1 ml of 1 × PBS followed by blocking with 0.5% BSA in 1 × PBS (80 *μ*l final volume) for 1 h at 21 °C. Immunolabelling of cleaved PARP or cleaved caspase-8 was performed at 21 °C for 1 h when using fluorescently labeled antibodies (Cleaved caspase-8 PE (cat. # 12602), Cleaved PARP Alexa 647 (cat. # 6987) and Cleaved PARP PE (cat. # 8978) from Cell Signaling) or incubated for 1 h with primary antibodies (Cleaved caspase-8 (cat. # 9496)), washed three times with 1 × PBS and incubated 1 h with fluorescently tagged secondary antibodies anti-rabbit Alexa 488 (cat. # A21206; Life Technologies). After staining, cells were washed twice in 1 ml of 0.5% BSA in 1 × PBS, re-suspended in 300 *μ*l of 0.5% BSA in 1 × PBS and immediately analyzed by FACS. Results presented in histograms were obtained from at least three independent biological replicates (independent cell cultures and treatments). At least 10 000 cells per individual experiment were analyzed.

### Surface levels of TRAIL receptors

Surface expression of TRAIL receptors was measured by flow cytometry. Cells were trypsinized, centrifuged at 1100 r.p.m. for 5 min, re-suspended in blocking solution (5% FCS in 1 × PBS) and counted. 10^5^ cells were incubated with blocking solution (5% FCS in 1 × PBS) for 1 h on ice and immunolabelled using antibodies recognizing DR4, DR5, DcR1 and DcR2 (catalog numbers under section 'Antibodies'). Appropriate IgGs were used as a control of nonspecific background fluorescence. Staining was performed for 1 h on ice in dark. Each sample was additionally labeled with DAPI to identify non-permeabilized viable cells. Expression of TRAIL receptors was assessed only in DAPI-negative cells.

### Immunoprecipitation

BJELR cells were either left untreated or treated with 1 *μ*g/ml TRAIL for 30 min (unless indicated otherwise) at regular adherent growing conditions (37 °C, 5% CO_2_). Cells were washed once with 1 × PBS in plate, then collected by scraping in 1 × PBS, centrifuged (at 1100 r.p.m. for 5 min at 4 °C) and lysed in 1 ml (for 2 × 10^7^ cells) of freshly prepared lysis buffer (30 mM Tris-HCl pH 7.4, 150 mM NaCl, 5 mM KCl, 10% glycerol, 2 mM EDTA pH 8.0) supplemented with EDTA-free protease inhibitor cocktail (Roche, cat. # 11873580001), phosphatase inhibitor (PhosStop, Roche, cat. # 04906845001) and 1% Triton-X100 (Sigma cat. # 93418). Lysates were incubated on ice for 30 min followed by 30 min centrifugation at 14 000 r.p.m. at 4 °C. Further, lysates were subjected for pre-clearing with Pure Proteome Protein G magnetic beads (Millipore, cat. LSKMAGG02) equilibrated in lysis buffer for 2 h at 4 °C (15 *μ*l beads for 1 ml of lysate). Binding of antibodies to the beads was performed in the proportion of 1 *μ*g of antibodies to 2 *μ*l beads, for 3 h at 21 °C and 1000 r.p.m. (Thermomixer Comfort Eppendorf, Austria). Next, lysates were quantified using Protein assay (BioRad, Germany, cat. # 500-0006), adjusted to same concentrations using lysis buffer and subjected to DR4, DR5, DcR2, TRAF2, RIPK1, caspase-8 immunoprecipitation using anti-DR4, anti-DR5, anti-DcR2, anti-TRAF2, anti-RIPK1, anti-caspase-8 antobodies (catalog number in section 'Antibodies'). Normal goat IgG or normal mouse IgG bound to Pure Proteome Protein G Magnetic Beads was used as nonspecific immunoprecipitation controls. Immunoprecipitations were performed overnight at 4 °C in a rotating shaker to ensure proper mixing of the sample. For caspase-8, DR4, DR5, DcR2, TRAF2 and RIPK1 IP immunoprecipitation we used 5 *μ*g of antibodies bound to 10 *μ*l beads for 2 × 10^7^ cells. After immunoprecipitation, beads were washed five times with ice-cold lysis buffer and eluted. Elution was performed using 1 × Laemmli buffer (two-fold concentrated buffer – 125 mM Tris-HCl pH 6.8; 4% SDS; 10% 2-mercaptoethanol; 10% glycerol – was diluted to one-fold using lysis buffer) supplemented with 20 mM DTT. Elution was done at 75 °C, 1000 r.p.m. for 15 min.

### Plasma membrane purification

Extraction of the plasma membrane fraction was performed using Plasma membrane protein extraction kit (BioVision, Exton, PA, USA, cat. K268-50). BJELR cells were either left untreated or treated with 1 *μ*g/ml TRAIL at regular growing conditions (37 °C, 5% CO_2_). Cells were washed in plate with 1 × PBS, collected in 1 × PBS, centrifuged (at 1100 r.p.m. for 5 min at 4 °C) and re-suspended in 2 ml (4 × 10^7^ cells per sample) of homogenization buffer supplemented with 8 *μ*l Protease Inhibitors Cocktail (reconstituted in 500 *μ*l of DMSO). Cells re-suspended in Homogenization buffer were homogenized in a Dounce homogenizer (Weaton 1 ml, loose pestle cat. # 0.089–0.14 mm) on ice. Cells were passed through the homogenizer 100 times resulting in a complete lysis (90% of cells) as determined by analyzing a fraction of the sample under the microscope. Further procedures were performed as indicated by the vendor. Briefly, obtained homogenate was transferred into a 1.5 ml microcentrifuge tube (2 ml of homogenate were divided into two tubes) and centrifuged at 700 × *g* for 10 min at 4 °C. Supernatant was transferred into a new microcentrifuge tube and centrifuged again at 700 × *g* for 10 min at 4 °C. Further, the supernatant was carefully collected, transferred into a new tube and centrifuged at 10 000 × *g* for 30 min at 4 °C. Supernatant represented the cytosol fraction, while the pellet contained proteins from the plasma membrane and membranes from cellular organelles. For plasma membrane purification, the pellet was re-suspended in 300 *μ*l of Upper Phase Solution followed by the addition of 300 *μ*l of Lower Phase Solution. The sample was well mixed and incubated on ice for 5 min. Further, the sample was centrifuged at 1000 × *g* for 5 min at 4 °C. The upper phase (purified plasma membrane fraction) was carefully transferred into a new tube and kept on ice. To maximize the yield of extraction, a second round of extraction was performed from the remaining lower phase by adding 150 *μ*l of Upper Phase Solution to the sample. As before, sample was mixed and centrifuged at 1000 × *g* for 5 min at 4 °C. The upper phase was carefully collected and combined with the upper phase obtained after the first extraction. Next, the combined upper phase was further purified by adding 150 *μ*l of Lower Phase Solution. Sample was mixed and centrifuged at 1000 × *g* for 5 min at 4 °C. The upper phase was carefully collected in 2 ml tubes and diluted in five volumes of distilled water (GIBCO, Gaithersburg, MD, USA, cat. # 10977-035), incubated for 10 min on ice and centrifuged at 15 000 × *g* for 10 min at 4 °C. The pellet containing the plasma membrane fraction was re-suspended in lysis buffer (30 mM Tris-HCl pH 7.4; 150 mM NaCl; 5 mM KCl; 10% glycerol; 2 mM EDTA pH 8.0) (0.5 ml for initial 2 × 10^7^ of cells) supplemented with EDTA-free protease inhibitor cocktail (Roche, Indianapolis, IN, USA, cat. # 11873580001), phosphatase inhibitor (PhosStop, Roche act. # 04906845001) and 1% Triton-X100 (Sigma cat. # 93418). To ensure proper reconstitution of the pellet, samples were incubated on ice for 20–30 min. Further, immunoprecipitation assays using plasma membrane fraction were performed as described in the 'Immunoprecipitation' section. For RIPK1, FADD and TRAF2 immunoprecipitation from the plasma membrane fraction 2.5 *μ*g of antibodies were bound to 5 *μ*l beads for 4 × 10^7^ cells whereas for DR4 and DR5 immunoprecipitations 5 *μ*g of antibodies bound to 10 *μ*l beads for 4 × 10^7^ cells were utilized. Anti-N-cadherin antibodies and anti-EEA1 antibodies were used as markers of plasma membrane and early endosomes (respectively) to verify the purity of the plasma membrane fraction.

### Western blot assay

Samples for western blot were washed three times in 1 × PBS and collected in a plate by adding RIPA buffer (10 mM Na_2_HPO_4_, 150 mM NaCl, 2 mM EDTA, 1% IGEPAL, 1% Deoxicolate Na, 0.1% SDS, 10 mM NaF) supplemented with EDTA-free protease inhibitor cocktail (Roche at. # 11873580001), phosphatase inhibitor (PhosStop, Roche at. # 04906845001). For western blots assessing the phosphorylated and total protein levels of Erk1/2, Akt, p38 and IκBα, apoptotic cells were removed by successive washings with 1 × PBS and remaining adherent cells (survivors) were collected. RIPA samples were lysed on ice for 30 min and centrifuged at 14 000 r.p.m. for 30 min at 4 °C. Supernatants were collected and stored at −80 °C or directly used for western blot assay. For that, samples were quantified using Protein assay (BioRad, cat. # 500-0006) and adjusted to the same concentration using RIPA buffer supplemented with EDTA-free protease inhibitor cocktail and phosphatase inhibitor. Samples were supplemented with loading buffer and incubated for 10 min at 100 °C (protein denaturation step). Proteins were separated by SDS-PAGE in the Min-PROTEAN Tetra Cell Run (BioRad cat. # 1658000EDU) at 120 V in 1 × Tris Glycine buffer (10 × Tris Glycine buffer (1 l: 30.2 g Tris Base, 188 g glycine, 50 ml SDS 20%) and transferred into nitrocellulose membranes (Amersham, UK, cat. # 10600033) in Criterion Blotter (BioRad cat. # 1704070) at 100 V for 45 min in 1 Tris Glycine buffer. Nitrocellulose membranes were stained with Ponceau S to control equal protein loading and transfer. Membranes were washed with 1 × PBS+0.1% Tween20 to remove Ponceau S staining and blocked using 5% non-fat milk reconstituted in 1 × PBS+0.1% Tween20 for 1 h at room temperature. Membranes were incubated with required primary antibodies either in 5% non-fat milk in 1 × PBS+0.1% Tween20 or in 5% BSA in 1 × PBS+0.1% Tween20 overnight at 4 °C in a head-to-head shaker. Next, membranes were washed three times in 1 × PBS+0.1% Tween20 and incubated with corresponding secondary peroxidase-conjugated antibodies for 1 h at room temperature, washed three times in 1 × PBS+0.1% Tween20 and immunoreactive bands were visualized by chemiluminescense using ECL western blotting substrate (Pierce, Waltham, MA, USA, cat. # 32209), SuperSignal West Pico Chemiluminescent substrate (Pierce, cat. # 34080) or Luminata Forte Western HRP Substrate (Millipore, cat. WBLUF0500) and exposed to Carestream Kodak BioMax MR film (cat. Z350389-50EA), Amersham Hyperfilm ECL (cat. # 28906835) or Amersham Hyperfilm MP (cat. # 28906842). Western blot exposures were scanned and processed in Adobe Photoshop CS4. Images presented in the manuscript correspond to one representative experiment out of at least two biological replicates. Western blot results were analyzed by double blind. Images were processed using Adobe Systems Incorporated (San Jose, CA, USA).

### Preparation of competent bacteria

A single colony of XL1 blue cells was used to inoculate 20 ml of LB medium. Bacteria was grown overnight at 37 °C 190 r.p.m. ('overnight pre-culture'). Ten milliliters of the 'overnight pre-culture' was used to inoculate 100 ml of pre-warmed (37 °C) LB medium that was incubated at 37 °C 190 r.p.m. until OD reached 0.48. Cultures were centrifuged for 10 min at 5000 r.p.m. at 4 °C, pellet was re-suspended in 30 ml of ice-cold TFBI buffer (for 100 ml: 1.2092 g RbCl_2_, 0.8094 g MnCl_2_, 0.2944 g CH_3_CO_2_K, 1 ml 1M CaCl_2_, 15 ml 100% glycerol, adjust pH to 5.8 using 0.2 M acetic acid), incubated on ice for 1–2 h and centrifuged again for 10 min at 5000 r.p.m. at 4 °C. Next, the pellet was re-suspended in 4 ml of ice-cold TFBII buffer (for 100 ml: 7.5 ml 1M CaCl_2_, 0.1209 g RbCl_2_, 0.2930 g MOPS, 15 ml 100% glycerol, adjust pH to 7.0), aliquots of 100 *μ*l were frozen in liquid nitrogen and stored at −80 °C.

### Bacteria transformation

One hundred nanograms of DNA were added to the competent bacteria and quickly mixed followed by 30 min incubation on ice. In order to optimize DNA uptake, samples were subjected to heat shock by incubation at 42 °C for 90 s followed by 5 min incubation on ice. Next, 500 *μ*l of LB buffer was added, material was transferred into 10 ml falcon and incubated at 37 °C for 30–45 min (with vigorous shaking). Finally, cells were plated on agar plates containing selection antibiotics and allowed to grow at 37 °C for 12–16 h.

### Reverse transcription reaction

RNA was isolated using Trizol reagent (Invitrogen 15596-026) following the manufacturer’s instructions. Samples were either stored at −80 °C (for a long-term storage) or used for reverse transcription (RT). For RT, 1 *μ*l of dNTPs (ThermoScientific R0141, R0151, R0161, R0171) was mixed with 1 *μ*l of 20 μM oligo dT, 3 *μ*g of RNA and H_2_O to a final volume of 12 *μ*l (reaction mix 1). The reaction mix 1 was heated at 65 °C for 5 min (denaturation step) and chilled on ice. In parallel, 4 *μ*l of Superscript buffer (SuperScript II Reverse Transcriptase Invitrogen, cat. # 18064-022) was mixed with 2 *μ*l of 0.1M DTT, 1 *μ*l of Ribonuclease inhibitor and 0.5 *μ*l of Superscript Reverse Transcriptase (SuperScript II Reverse Transcriptase Invitrogen, cat. # 18064-022) (reaction mix 2). The reaction mix 1 was well mixed with the reaction mix 2 and incubated at 44 °C for 1 h (reverse transcription) followed by heat inactivation (70 °C for 15 min).

### PCR and cloning

Primers used for DR4 cDNA amplification were the following: pair 1 – forward atggcgccaccaccagctagag and reverse gtcactccagggcgtacaat; pair 2 – forward ggaactttccggaatgacaa and reverse tcactccaaggacacggcagagc. PCR Mix 1 and Mix 2 were prepared as follows. One microliter of dNTPs (10 mM each) was mixed with 1 *μ*l of 20 *μ*M Forward primer, 1 *μ*l of 20 *μ*M Reverse primer, 5 *μ*l of cDNA (1/10 dilution of the RT product) and H_2_O to the final volume of 25 *μ*l (mix 1). In parallel 5 *μ*l of high fidelity polymerase buffer (Roche, cat. # 14676700) was mixed with 19.25 *μ*l of H_2_O and 0.75 *μ*l of high fidelity polymerase (Roche, cat. # 14528824) (mix 2). Mix 1 was combined with Mix 2 and subjected to PCR. Efficiency of PCR was verified by analyzing PCR products by gel electrophoresis (2% agarose gel in TBE buffer supplemented with ethidium bromide). The required band was purified from the gel using Minielute gel extraction Kit (Qiagen, cat. # 28604). Purified PCR fragments were cloned into the pGEM-T vector (pGEM-T easy vector system Promega, cat. # A1360). The following day competent XL1 blue bacteria were transformed with the constructs as described in the 'Bacteria transformation' section. Agar plates were supplemented with 100 *μ*l of LB, 10 *μ*l of XGal (100 mg/ml) and 10 *μ*l of IPTG (1M). After overnight growth at 37 °C, white colonies were selected for liquid culture (1 colony for 3.5 ml of LB medium supplemented with ampicillin). Plasmid purification was performed using NucleoSpin Plasmid Kit (Macherey-Nagel, cat. # 740588.50). Sequencing of DR4 cDNA was done in the AGTC Biotech utilizing T7 and SP6 primers.

### qPCR

cDNA was diluted 10 times prior to real time qPCR. Briefly, 0.2 *μ*l (100*μ*M) of forward primer was mixed with 0.2 *μ* (100 *μ*M) of reverse primer, 10 *μ*l of Qiamix (Qiagen, cat. 204141) and 4.5 *μ*l of H_2_O. This mix was placed into 96-well qPCR plate and supplemented with 5 *μ*l of previously diluted cDNAs. Two independent biological replicates (three technical replicates each) were performed for each condition. Glyceraldehyde 3-phosphate dehydrogenase levels were used for normalization.


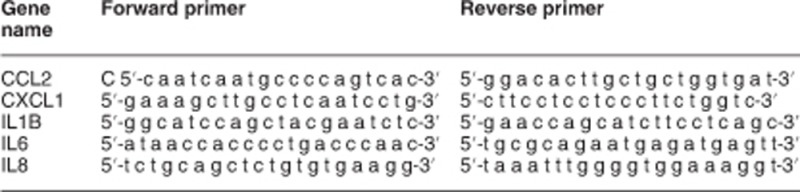


### Statistical analysis

Results represented in the histograms show the mean value±standard deviation (S.D.) of at least three independent biological replicates. Statistical significance was calculated by applying two-tailed, unpaired Student’s *t*-test, ***P-value <0.0005, **P-value <0.005, *P-value <0.05.
