# Supplementary material for: Dual role of DR5 in death and survival signaling leads to TRAIL resistance in cancer cells
Source: Cell Death Dis. 2017 Aug 31;8(8):e3025–. doi: 10.1038/cddis.2017.423 (PMC5596601; doi:10.1038/cddis.2017.423)
Supplement: Supplementary Information [file cddis2017423x1.docx]

**Dual role of DR5/TAILR2 in death and survival signaling leads to TRAIL resistance in cancer cells**

**Yelyzaveta Shlyakhtina^1,2^, Valeria Pavet^1,2,*^ and Hinrich Gronemeyer^1,*^**

^1^Institut de Génétique et de Biologie Moléculaire et Cellulaire (IGBMC), Equipe Labellisée Ligue Contre le Cancer, Centre National de la Recherche Scientifique UMR 7104, Institut National de la Santé et de la Recherche Médicale U964, University of Strasbourg, Illkirch, France.^2^Equal contribution. ^*^Correspondence: hg@igbmc.fr and [vpavet@igbmc.fr](mailto:vpavet@igbmc.fr)

**Supplementary Information**

**Supplementary Figure Legends**

**Supplementary figure 1| (a)** Contour plot assessing the percentage of cells displaying positive labelling for cleaved PARP in populations of 8 independent BJELR subclones left unchallenged (control) or treated for 6h with TRAIL (TRAIL). Subpopulations displaying low (“1”) and high (“2”) levels of cleaved PARP are indicated. The percentage of cells displaying high level of cleaved PARP is shown (“2”). Depicted image for each indicated clone corresponds to one representative experiment out of at least two independent biological replicates. **(b-c)** Western blots displaying total and phosphorylated protein levels of Erk1/2, Akt, p38 and IκBα (regulator of NF-κB signaling) in two independent BJELR subclones (Clone 9E and Clone 8A) left unchallenged (0) or upon TRAIL treatment for the indicated time points. Surviving (adherent) cells were used for protein extraction whereas apoptotic cells were removed by extensive washings. Depicted images correspond to one representative experiment out of three independent biological replicates. α- Tubulin, loading control. **(d)** Western blots displaying total and phopshorylated levels of indicated proteins in cells treated for 1h with U0126 (MEK1/2 inhibitor), LY294002 (PI3 Kinase inhibitor), PD169316 (p38 inhibitor) or Bay 11-7082 (IKK inhibitor) as compared to vehicle (DMSO). Depicted images correspond to one representative experiment out of three independent biological replicates. α- Tubulin, loading control.

**Supplementary Figure 2| (a)** Efficiency of DR4, DR5 and DcR2 knock down using pooled siRNAs**.** Western blots displaying DR4, DR5 or DcR2 protein levels in BJELR cells transfected with pooled siRNAs targeting *DR4* (siDR4), *DR5* (siDR5), *DcR2* (siDcR2) mRNAs or non-targeting scramble siRNAs (scr). Depicted images correspond to one representative experiment out of three independent biological replicates. β-Actin, loading control. **(b)** Efficiency of DR4, DR5 and DcR2 knock down using individual siRNA**.** Western blots displaying DR4, DR5 or DcR2 protein levels in BJELR cells transfected with individual siRNAs targeting *DR4* (siDR4-8 and siDR4-9), *DR5* (siDR5-6, siDR5-7 or siDR5-8) or *DcR2* (siDcR2-14, siDcR2-15) mRNAs or non-targeting scramble siRNAs (scr) at 48h post transfection. Images from one representative experiment out of three independent biological replicates are shown. α- Tubulin, loading control. **(c-f)** Role of DR4, DR5 and DcR2 in the triggering of apoptosis. Dot plots from flow cytometry assays assessing the percentage of cells displaying high levels of cleaved PARP in naïve (control) or after 3h of TRAIL treatment (TRAIL) in BJELR cells transfected with non-targeting scramble siRNAs (scramble; **c**), individual siRNAs targeting *DR4* (DR4 si8, DR4 si9; **d**), *DR5* (DR5 si6, DR5 si7, DR5 si8; **e**) or *DcR2* (DcR2 si14, DcR2 si15; **f**) mRNAs. Images from one representative experiment out of three independent biological replicates are shown. Percentage of cells displaying high level of PARP cleavage is indicated.

**Supplementary Figure 3| (a)** Western blots displaying DR5 or DcR2 protein levels in DR5 knock out (DR5 KO), DcR2 knock out (DcR2 KO) or control BJELR cells. **(b)** Western blots displaying FADD, caspase-8, RIPK1, TRAF2 or cFlip protein levels in BJELR cells transfected with siRNAs targeting *FADD* (siFADD), *caspase-8* (sicasp8), *RIPK1* (siRIPK1), *cFlip* (sicFlip), *TRAF2* (siTRAF2) mRNAs or non-targeting scramble siRNAs (scr). **(c)** Western blots displaying RIPK1 and TRAF2 protein levels in BJELR cells transfected with siRNAs targeting *RIPK1* (siRIPK1), *TRAF2* (siTRAF2), or double transfected with a mix targeting *RIPK1* and *TRAF2* (siRIPK1 + siTRAF2) mRNAs or non-targeting scramble siRNAs (scr). **(d)** Western blots displaying DR5, RIPK1, TRAF2 protein levels in BJELR cells transfected with siRNAs targeting *DR5* (siDR5), *RIPK1* (siRIPK1), *TRAF2* (siTRAF2), a mix targeting *RIPK1* and *DR5* (siRIPK1 + siDR5) or *TRAF2* and *DR5* (siTRAF2 + siDR5) mRNAs or non-targeting scramble siRNAs (scr). **(a-d)** Representative images of knock down efficiency observed at at 48 h post transfection are displayed. α- Tubulin, loading control.

**Supplementary Figure 4| (a)** Uncropped gels corresponding to Figure 4e. Western blots displaying total and phosphorylated protein levels of Erk1/2 and Akt in naïve or TRAIL treated BJELR cells previously transfected with siRNAs targeting *cFlip* (sicFlip) mRNA or non-targeting scramble siRNAs (scr).

**Supplementary Figure 5| (a)** qPCR analysis of CCL2, CXCL1, IL1B, IL6 and IL8 mRNA levels in TRAF2 (siTRAF2) and RIPK1 (siRIPK1) depleted BJELR cells compared to scr transfected cells (ctrol), normalized to Glyceraldehyde 3-phosphate dehydrogenase (GAPDH) levels. Histograms represent the mean fold change in TRAIL treated samples (6 h treatment) as compared to non-treated cells for scr (control), siTRAF2 and siRIPK1 transfected cells (48h post transfection) +/- SD of two independent biological (three technical replicates each). Western blots displaying RIPK1 and TRAF2 protein levels in BJELR cells transfected with siRNAs targeting *RIPK1* (siRIPK1) and *TRAF2* (siTRAF2) or non-targeting scramble siRNAs (“scr”). Representative images of knock down efficiency are displayed. α- Tubulin, loading control. **(b)** qPCR analysis of CCL2, CXCL1, IL1B, IL6 and IL8 mRNA levels in DcR2 knock out BJELR cells (DcR2 KO) compared to wild type cells (ctrol), normalized to Glyceraldehyde 3-phosphate dehydrogenase (GAPDH) levels. Histograms represent the mean fold change in TRAIL treated samples (6 h treatment) as compared to non-treated cells for DcR2 knock out (DcR2 KO) and wild type (WT) cells +/- SD of two independent biological (three technical replicates each). ***P value*<0.005, **P value*<0.05.

**Supplementary Figure 6| (a)** Uncropped image for Figure 5d. Western blots displaying total protein and phosphorylation levels of Erk1/2, Akt, p38 and IκBα in BJELR cells transfected either with siRNAs targeting *DR4* and *DcR2* (siDR4+siDcR2) mRNAs or non-targeting scramble siRNAs (scr) further left untreated (0) or challenged with TRAIL (1μg/ml). **(b)** Western blots displaying DR4, DR5 and DcR2 protein levels in BJELR cells transfected with siRNAs targeting *DR4* (pool, siDR4), *DR5* (pool, siDR5), *DcR2* (pool, siDcR2) a mix targeting both DR4 and DcR2 (siDR4+siDcR2) or non-targeting scramble siRNAs (“scr”). Representative images of knock down efficiency are displayed. α- Tubulin, loading control.

**Supplementary Figure 7|** Western blots displaying total and phosphorylated protein levels of Erk1/2, Akt, p38 and IκBα in HeLA **(a)** and HCT116 cells **(c)** challenged with DR5 selective TRAIL-mimetic peptide M1D (10 μM) as compared to controls (0). Images correspond to one representative experiment out of three independent biological replicates. α- Tubulin, loading control. Contour plot from flow cytometry assays assessing cleaved PARP immunolabelling in populations of naïve (control) or M1d treated HeLa (b) and HCT116 (d) cells. Subpopulations displaying low (1; surviving cells) and high (2; apoptotic cells, percentage is depicted) levels of PARP cleavage are indicated.**(e-f)** Surface levels of Death Receptor 5 (anti-DR5-PE) and Death Receptor 4 (anti-DR4 PE) in HeLA **(e)** and HCT116 **(f)** cells. Isotypic IgG1PE labeling was used as background fluorescence control.

**Supplementary Figure 8| (a-h)** Efficiency of the fractionation of cellular proteins into plasma membrane, endosomes and cytosol fractions. **(a)** Purity control for Figure 6 a. BJELR cells were either left untreated (-) or treated with 1μg/ml TRAIL during 30 min (+) and further lysed using either DISC lysis buffer (whole cell lysate; WCL) of subjected to cellular fractionation. Distribution of canonical DISC components as well as RIPK1 and TRAF2 in whole cell lysate and different fractions (plasma membrane, endosomes, cytosol) was assessed by Western blot. N-cadherin was used as a marker of plasma membrane fraction. EEA1 was used as a marker of endosomes fraction. **(b-h)** Purification controls of extracted plasma membrane as assessed by levels of EEA1 endosome marker in purified plasma membrane as compared to cytosol fraction. **(b)** Control for Figure 6 b. **(c)** Control for Figure 6 c. **(d)** Control for Figure 6 d-e. **(e)** Control for Figure 7a. **(f)** Control for Figure 7b. **(g)** Control for Figure 7c. **(h)** Control for Figure 7d.

**Supplementary Figure 9| (a)** NEMO interacts with RIPK1 at the plasma membrane. Western blots assessing the co-immunoprecipitation of NEMO with RIPK1 (RIPK1 immunoprecipitation: RIPK1 IP) in plasma membrane fractions obtained from naïve BJELR cells (-) or cells challenged with 1μg/ml TRAIL (+) at the indicated time points. FADD co-immunoprecipitation was analyzed as a positive control of the formation of TRAIL-induced signaling complex(es) in response to TRAIL challenge. N-Cdh is shown as plasma membrane marker. **(b)** Purification controls of extracted plasma membrane corresponding to the experiment displayed in **a** as assessed by levels of EEA1 endosome marker in purified plasma membrane as compared to cytosol fraction.\

**Supplementary Figure 10| (a)** Alignment of the intracellular domain of DR4 expressed in BJELR cells with the reference sequence.
