# Supplementary figures and images for: Dual role of DR5 in death and survival signaling leads to TRAIL resistance in cancer cells
Source: Cell Death Dis. 2017 Aug 31;8(8):e3025–. doi: 10.1038/cddis.2017.423 (PMC5596601; doi:10.1038/cddis.2017.423)

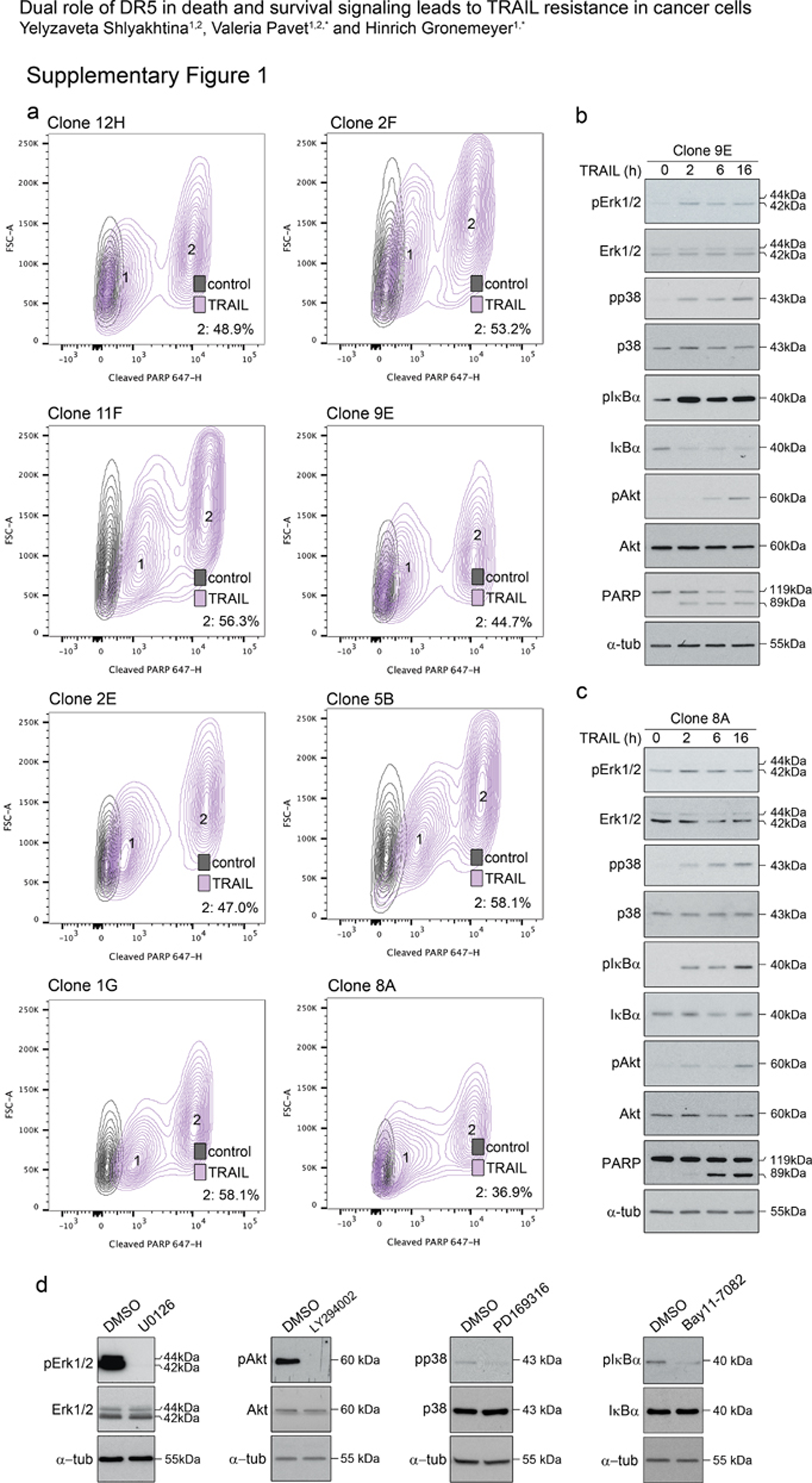

Supplement: Supplementary Figure 1 [file cddis2017423x2.tif]

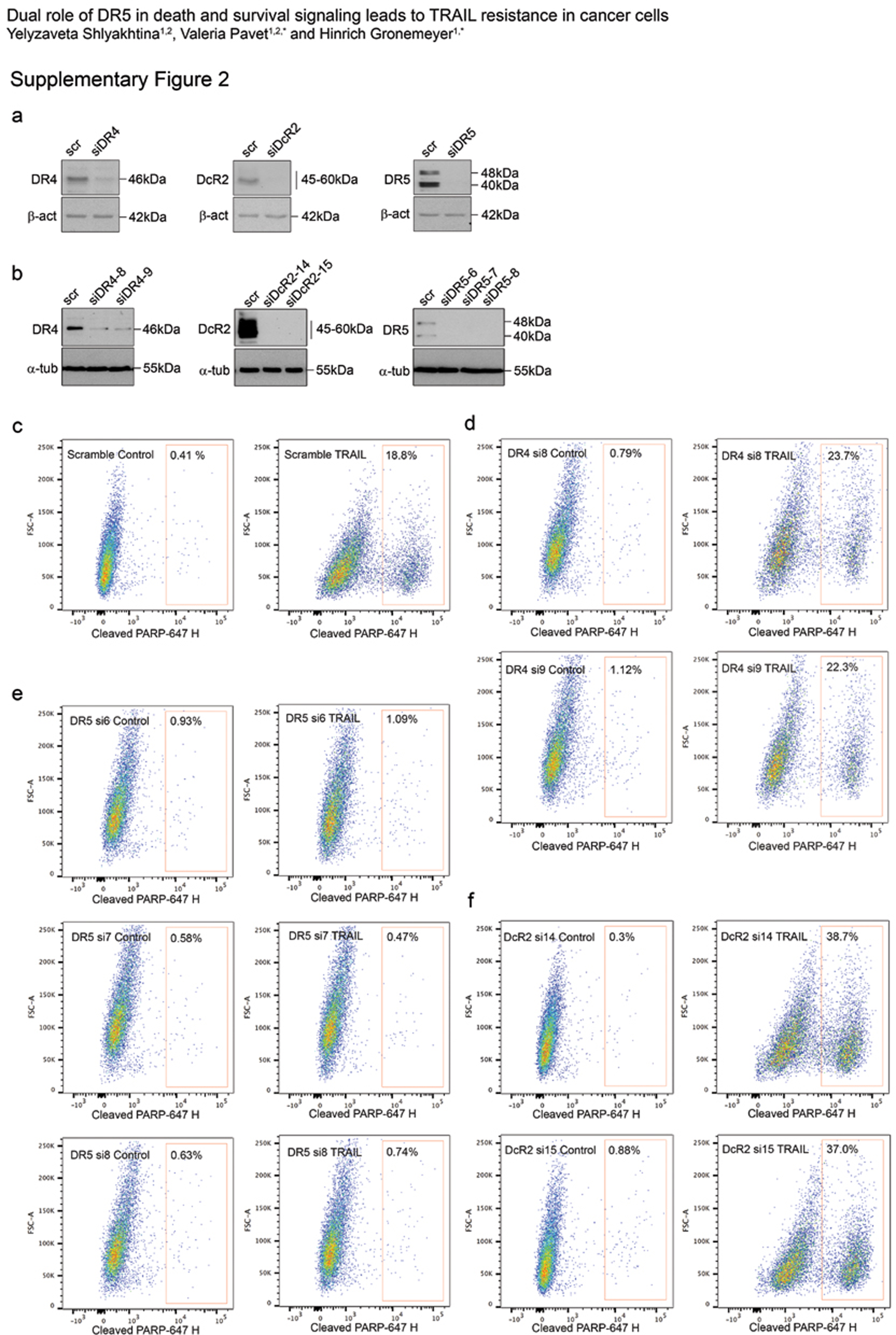

Supplement: Supplementary Figure 2 [file cddis2017423x3.tif]

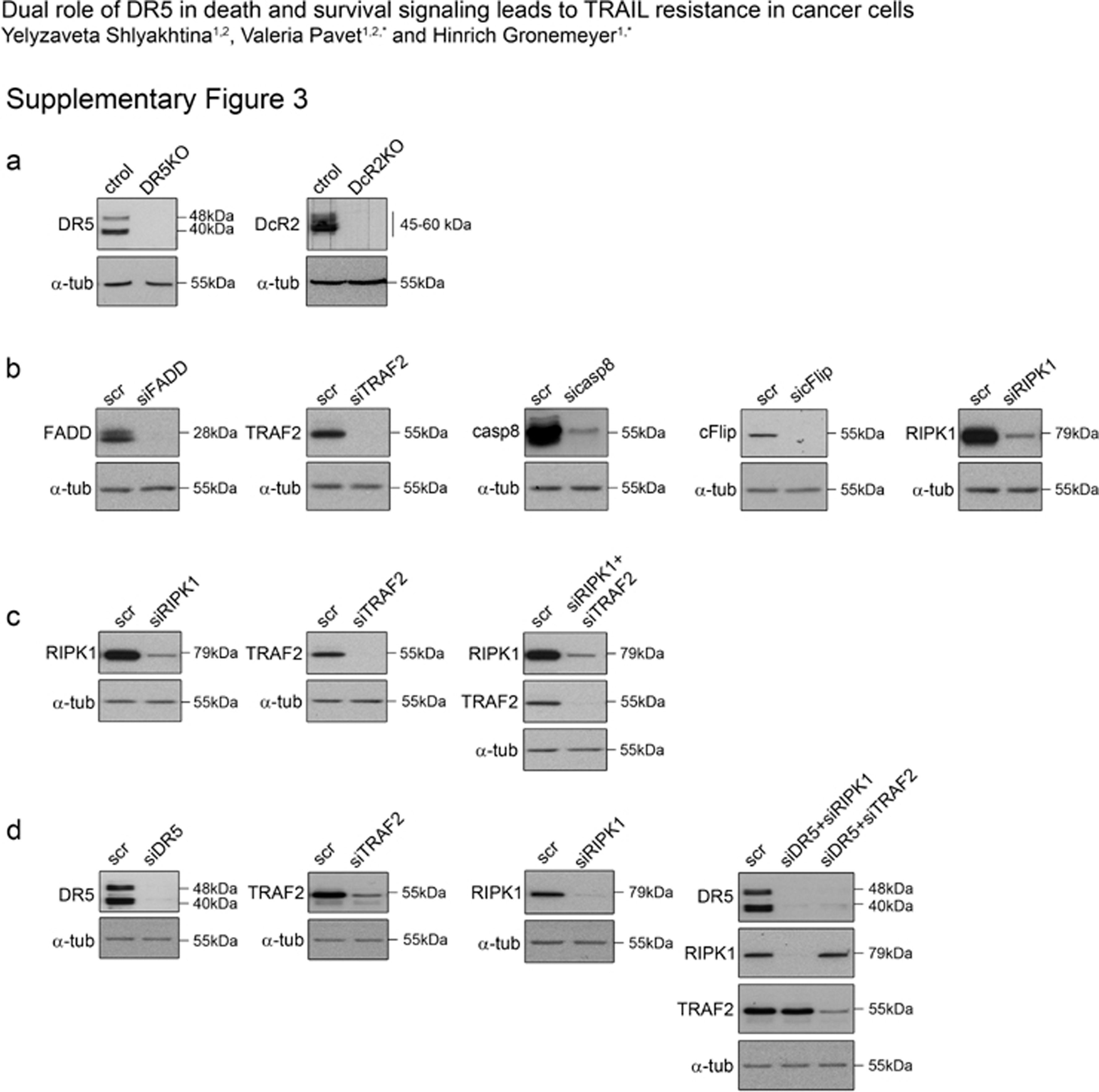

Supplement: Supplementary Figure 3 [file cddis2017423x4.tif]

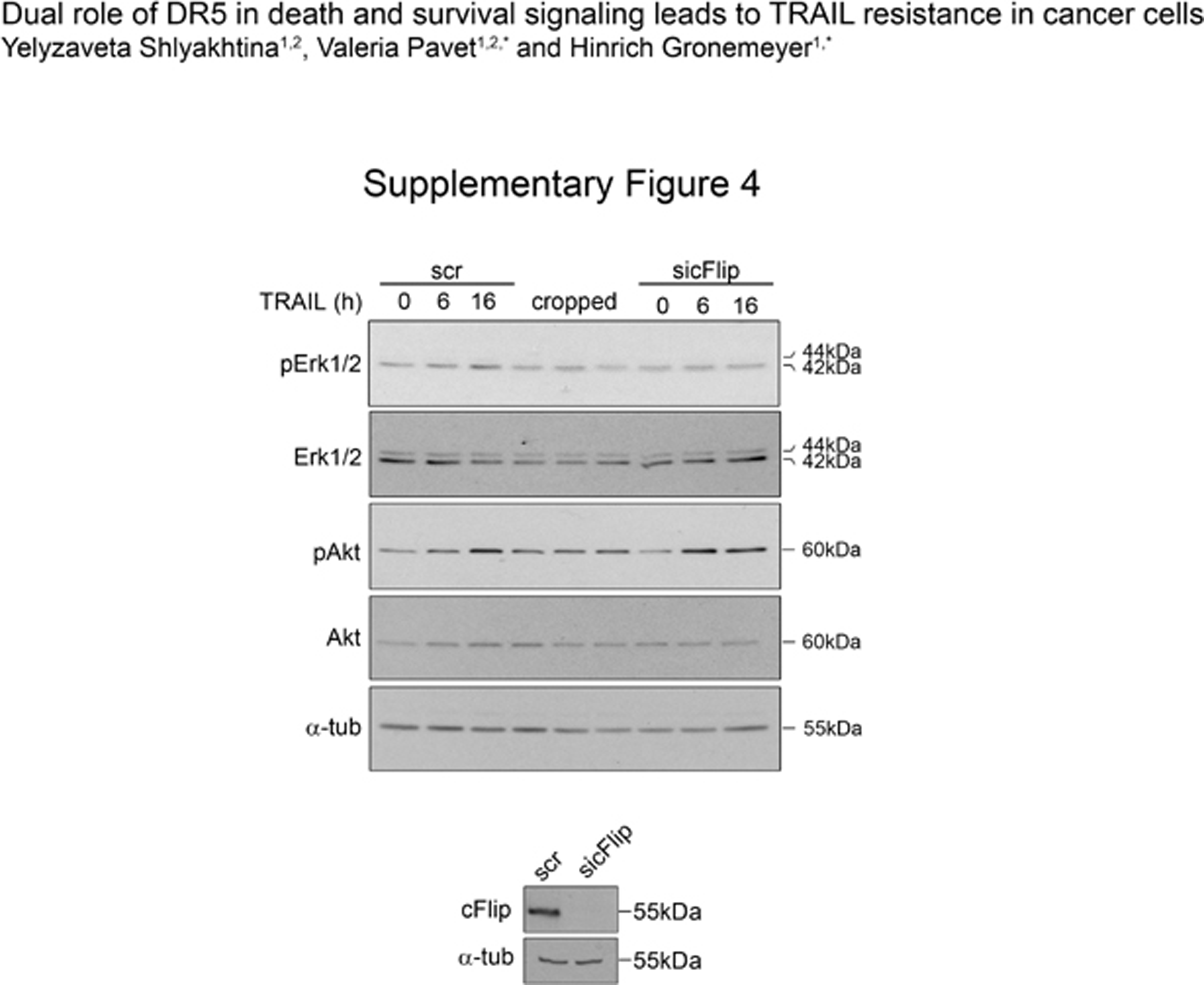

Supplement: Supplementary Figure 4 [file cddis2017423x5.tif]

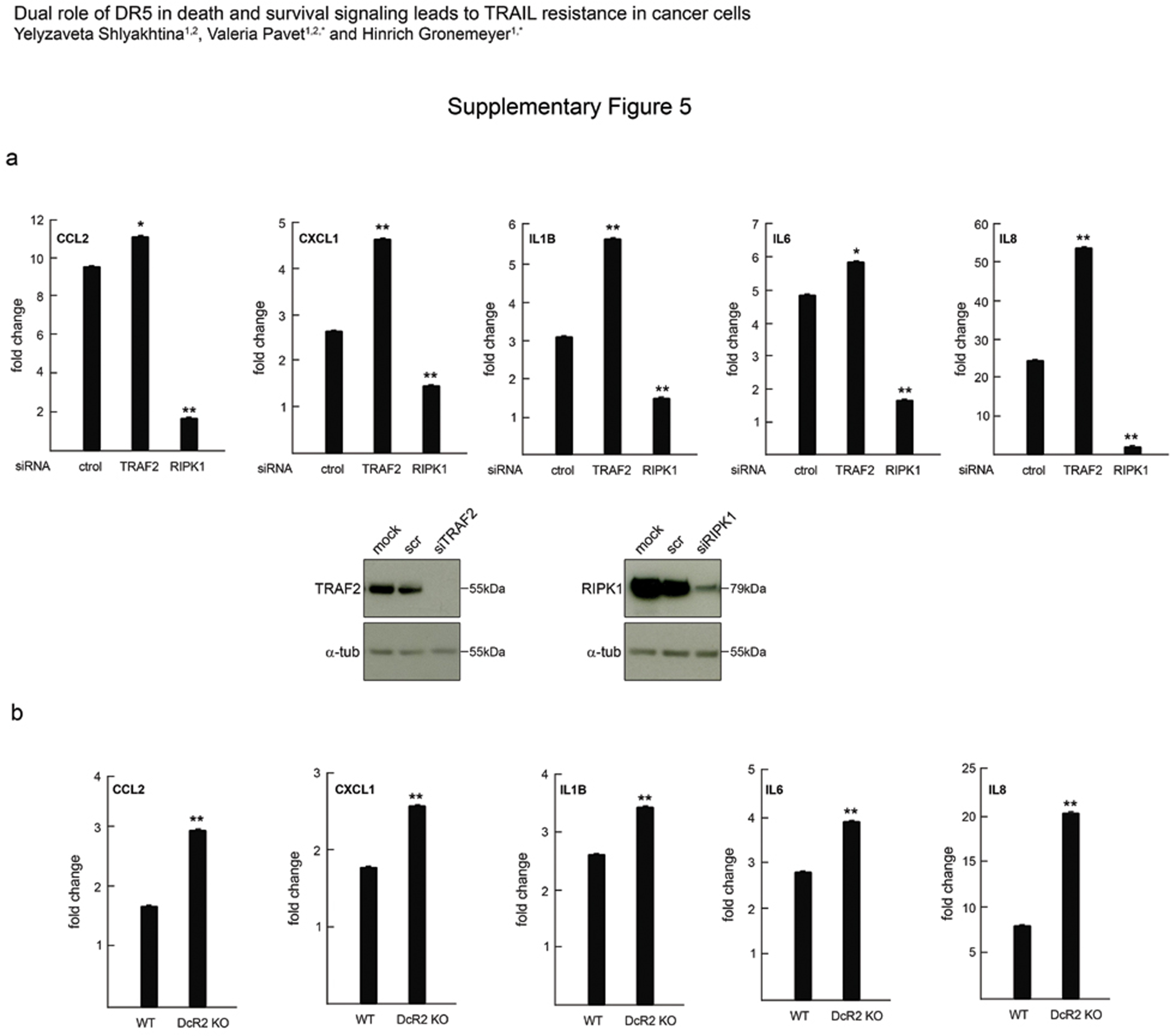

Supplement: Supplementary Figure 5 [file cddis2017423x6.tif]

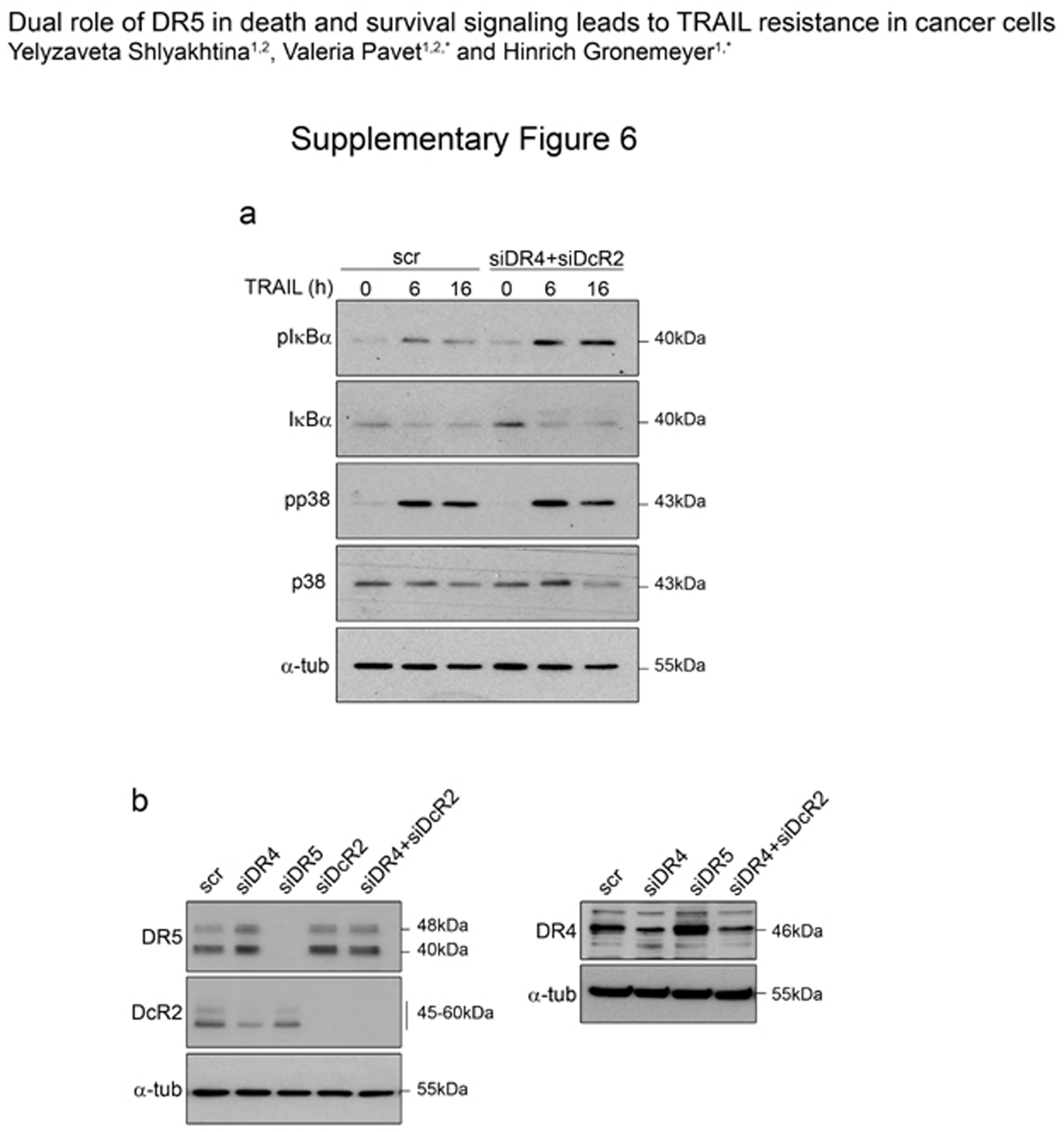

Supplement: Supplementary Figure 6 [file cddis2017423x7.tif]

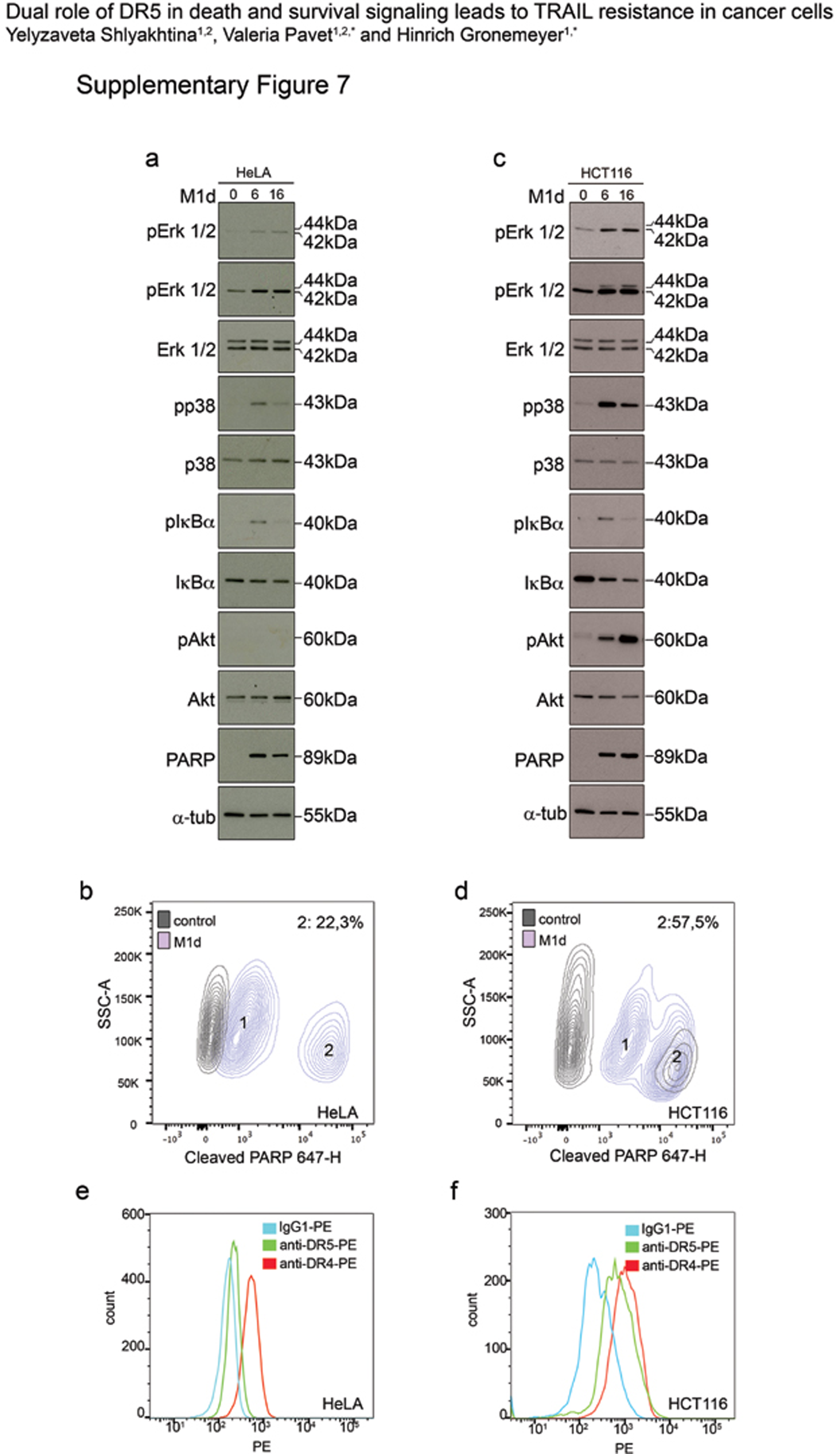

Supplement: Supplementary Figure 7 [file cddis2017423x8.tif]

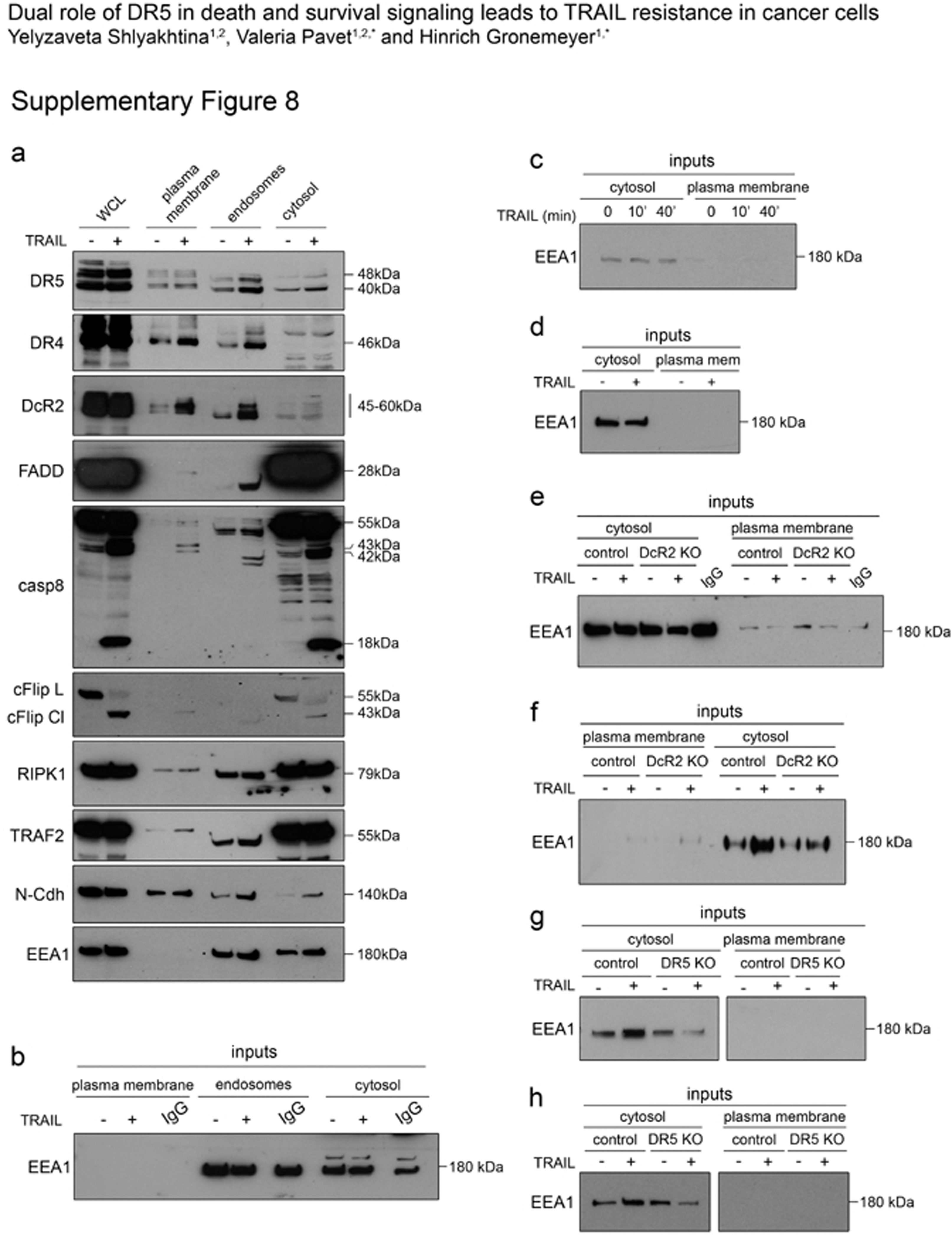

Supplement: Supplementary Figure 8 [file cddis2017423x9.tif]

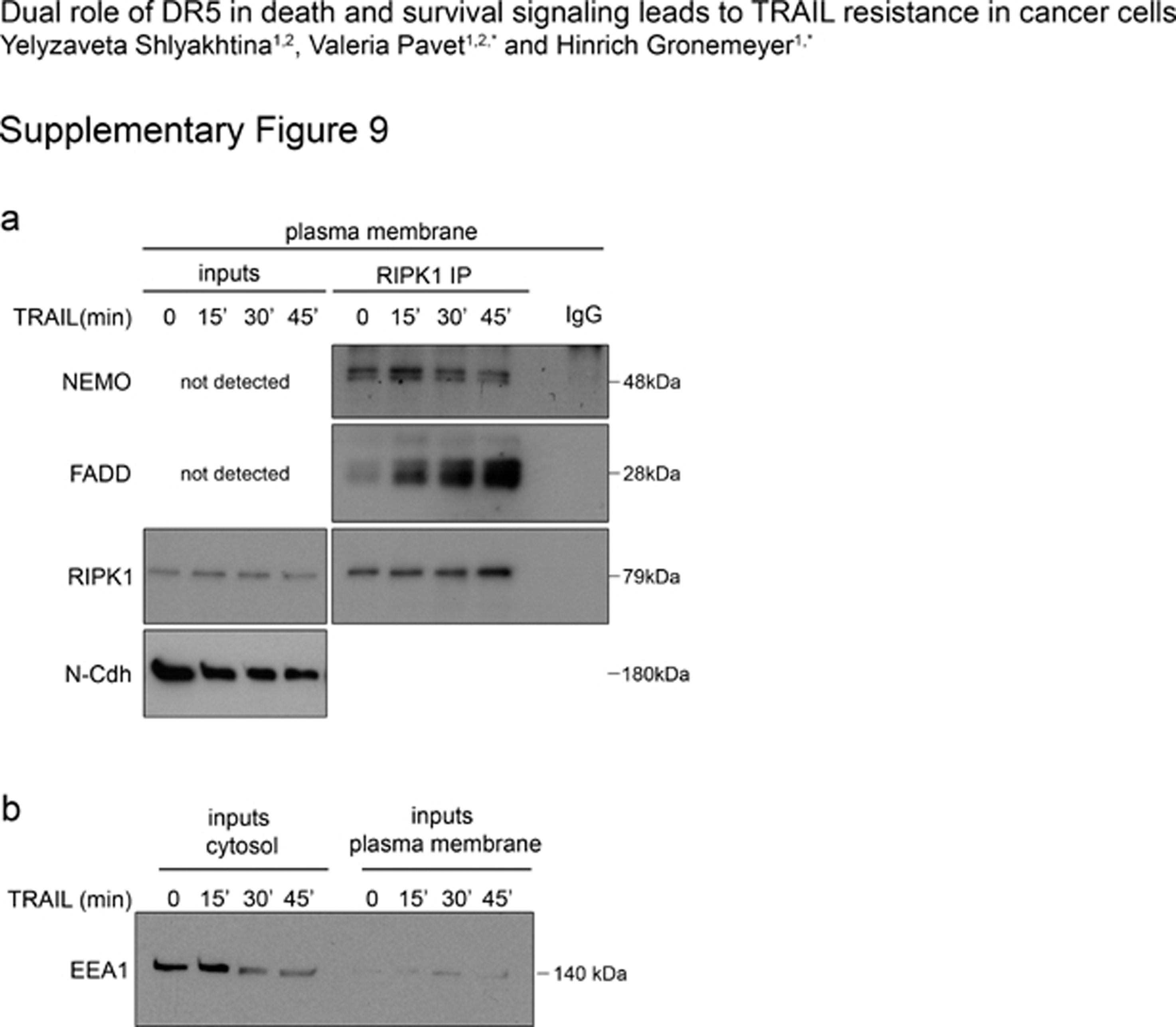

Supplement: Supplementary Figure 9 [file cddis2017423x10.tif]

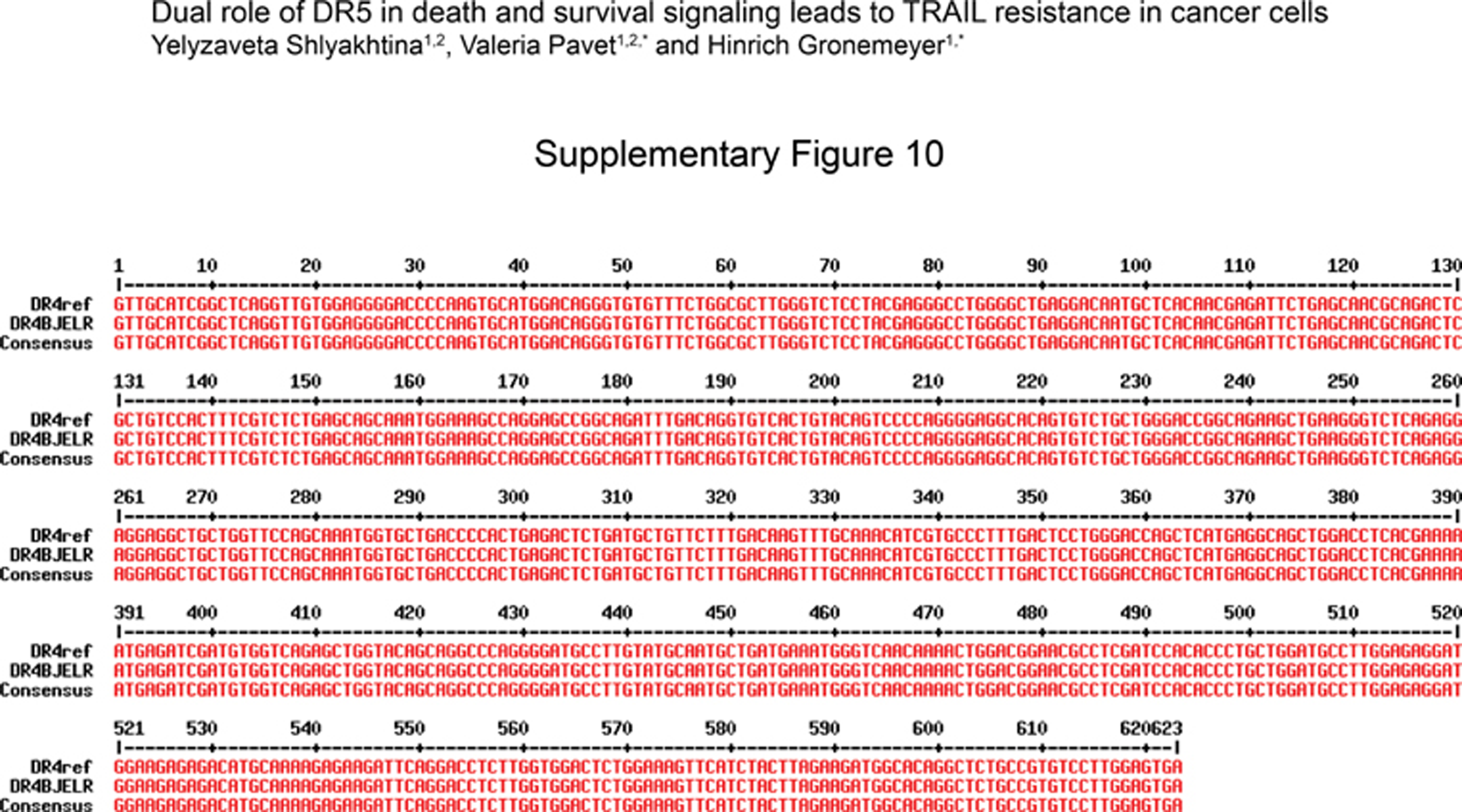

Supplement: Supplementary Figure 10 [file cddis2017423x11.tif]
